# Validation of the open biological negative image set for a Portuguese population: Comparing Japanese and Portuguese samples and an exploration of low-order visual properties of the stimuli

**DOI:** 10.3758/s13428-023-02090-9

**Published:** 2023-03-07

**Authors:** Nuno Gomes, Miguel F. Benrós, Gün R. Semin

**Affiliations:** 1 William James Center for Research, ISPA – Instituto Universitário, Rua Jardim do Tabaco 34, 1149-041 Lisbon, Portugal; 2 Faculty of Social and Behavioral Sciences, Utrecht University, Utrecht, The Netherlands

**Keywords:** Open biological negative image set, Disgust, Fear, Happiness, European, Portuguese

## Abstract

Recently, Shirai and Watanabe *Royal Society Open Science, 9*(1), 211128 (2022) developed OBNIS (Open Biological Negative Image Set), a comprehensive database containing images (primarily animals but also fruits, mushrooms, and vegetables) that visually elicit disgust, fear, or neither. OBNIS was initially validated for a Japanese population. In this article, we validated the color version of OBNIS for a Portuguese population. In study 1, the methodology of the original article was used. This allowed direct comparisons between the Portuguese and Japanese populations. Aside from a few emotional classification mismatches between disgust, fear, or neither-related images, we found that arousal and valence relate distinctively in both populations. In contrast to the Japanese sample, the Portuguese reported increased arousal for more positive valenced stimuli, suggesting that OBNIS images elicit positive emotions in the Portuguese population. These results showed important cross-cultural differences regarding OBNIS. In study 2, a methodological change was introduced: instead of the three classification options used originally (fear, disgust, or neither), six basic emotions were used (fear, disgust, sadness, surprise, anger, happiness), and a “neither” option, to confirm whether some of the originally “neither-related” images are associated with positive emotions (happiness). Additionally, the low-order visual properties of images (luminosity, contrast, chromatic complexity, and spatial frequency distribution) were explored due to their important role in emotion-related research. A fourth image group associated with happiness was found in the Portuguese sample. Moreover, image groups present differences regarding the low-order visual characteristics, which are correlated with arousal and valence ratings, highlighting the importance of controlling such characteristics in emotion-related research.

## Introduction

Research on behavior and emotions has increased exponentially in the last decades. Consequently, many efforts were made to develop standardized tools that allow researchers to study emotions in controlled laboratory settings (Davidson & Cacioppo, 1992). Examples of these tools are stimuli sets developed and used in affective and cognitive research (e.g., Open Affective Standardized Image Set; Kurdi et al., 2017; International Affective Picture System; Lang et al., 2005). Recently, Shirai and Watanabe (2022) developed a new stimulus set (Open Biological Negative Image Set; OBNIS) containing images categorized as disgusting, fearful, or neither. OBNIS was initially validated for a sample of the Japanese population.

Nevertheless, comparisons made using other image sets show important differences (especially regarding arousal and valence ratings) between Eastern and Western samples and even among distinct Western countries (e.g., Soares et al., 2015). In the present article, two studies were conducted to validate the color images on OBNIS for a Portuguese population. Potential similarities and differences between the Japanese and Portuguese populations were examined. We also explored low-order visual properties that are relevant parameters for studying affective reactions to emotion-related visual stimuli (i.e., luminosity, contrast, spatial frequency distribution, and chromatic complexity; e.g., Carretié et al., 2019). Below, we briefly discuss the relevance of datasets like OBNIS (i.e., involving fear and disgust-related stimuli) for psychology research, turning to the present studies' characteristics.

Fear and disgust are two functionally adaptive emotional states that facilitate avoiding potentially dangerous events (Öhman & Mineka, 2001; Olatunji & Sawchuk, 2005). Although both are threat-related, they are associated with distinct types of danger, presenting fundamental differences. Fear is related to impeding dangers (e.g., animal attacks; Gray, 1987), increasing sensory acquisition (Susskind et al., 2008), and facilitating quick avoidance responses (e.g., fight or flight responses; Öhman & Mineka, 2001). On the other hand, disgust is related to contamination (e.g., contact with animals carrying diseases; Rozin & Fallon, 1987), triggering more passive avoidance responses (e.g., reduced sensory acquisition; Susskind et al., 2008). However, despite the differences between fear and disgust, these two emotional states (or, more specifically, problems in regulating them) are thought to be behind many anxiety-related disorders (e.g., phobias; Öhman & Mineka, 2001; Olatunji & Sawchuk, 2005), being sometimes difficult to understand the relative contribution of each of them to a given disorder (e.g., Cisler et al., 2009). Hence, the study of fear and disgust and the resulting need to develop stimulus sets that allow inducing and distinguishing between these two emotional states are important contributions to research in basic and applied psychology.

Interestingly, animal stimuli appear to be the ideal candidates to integrate a stimulus set containing fear and disgust elicitors (e.g., Matchett & Davey, 1991; Öhman & Mineka, 2001; Webb & Davey, 1992) because they are associated with major common phobias (Becker et al., 2007). There is a recent growing interest in distinguishing between animals that trigger fear and disgust states in human species (e.g., Polák et al., 2020; Staňková et al., 2021), but most of the existing databases lack focused sets of animal stimuli (Grimaldos et al., 2021). Only a few attempts were made to overcome this limitation, such as the GAPED (Geneva Affective Picture Database; Dan-Glauser & Scherer, 2011) or the recent ASSAI (Affective Standardized Set of Animal Images; Grimaldos et al., 2021). Both include large sets of distinct animal species. Nevertheless, these databases were put together to assess general dimensions such as valence and arousal and not distinguish between stimuli evoking distinct emotional states.

To overcome this limitation of previous stimulus sets, Shirai and Watanabe (2022) created and validated the OBNIS. OBNIS consists of 200 open-source, no-background biological images (primarily animals but also fruits, plants, and mushrooms) that were classified (their colored and greyscale versions) as fearful, disgusting, or neither, by a Japanese sample. Ratings of arousal and valence were also collected for each image. However, following the observed data regarding other stimulus sets (e.g., the International Affective Picture System – IAPS; Lang et al., 2005), cultures seem to have an important influence on the way individuals perceive, feel, and express emotions (e.g., Soares et al., 2015; see also Yik et al., 2022). For instance, Indian participants rated pictures from IAPS with higher levels of arousal when compared to American participants (Lohani et al., 2013). Moreover, in most of the Western samples (e.g., American, Spanish, Hungarian, Portuguese), valence and arousal tend to have a quadratic (V-shaped) relation when IAPS stimuli are considered (e.g., Soares et al., 2015). However, Indian data showed no significant quadratic relation between these two emotion-related dimensions (Lohani et al., 2013). Contrary to the evidence in most Western validations, Indian participants showed a stronger relation between valence and arousal for positive compared to negative stimuli (Lohani et al., 2013). Altogether, these results suggest important cross-cultural differences in the normative data for affective visual stimuli image sets – particularly in comparisons between Western and Eastern samples. Hence, obtaining normative values for a Western population regarding the stimuli in OBNIS constitutes a necessary step to make it accessible to researchers in this field.

Thus, our aim with this article was to validate the colored version of the images constituting OBNIS for a Portuguese population. In the first study, we replicated the methodology used by Shirai and Watanabe (2022), allowing direct comparisons between the results obtained for the Portuguese population and the Japanese data. Not only the classification in the distinct emotional descriptors (fear, disgust, or neither) is inspected, but we also explored the relationship between valence and arousal ratings for both populations. In a second study, we introduce an important methodological change to the method used in the original article and our first study: instead of three classification options (disgust, fear, or neither), we used the six basic emotions as defined by Ekman (1992) (i.e., fear, disgust, sadness, surprise, anger, happiness) plus a “neither” option. In such a way, we can assess whether some of the images are associated with emotional states other than fear and disgust or are considered neutral.

Moreover, research has shown that low-order visual properties (i.e., physical characteristics) such as luminosity, contrast, chromatic complexity, and spatial frequency distribution (SF) are critical in affective sciences, modulating, attentional, behavioral, and neural responses to the images (Carretié et al., 2019; Redies et al., 2020). For instance, brighter images tend to be evaluated in general as more positive (Lakens et al., 2013) and brighter unpleasant images seem to elicit lower amplitudes of delta responses in electroencephalography records (Kurt et al., 2017). Furthermore, increasing the contrast of a target visual stimuli relative to the background increases fixations and detection alike in attention-related paradigms (t Hart et al., 2013). Regarding chromatic complexity, more complex (emotional) images seem to trigger more event-related potentials in visual (occipital) areas (Bradley et al., 2007). Additionally, Delplanque et al. (2007) showed that a selection of emotional scenes has a greater spectral density in low-spatial frequency bands than neutral ones. In the same vein, research has shown that the spatial frequency content of the stimuli affects the processing of emotion-related stimuli. For instance, the rapid detection of snakes (a well-known fear-related stimulus; e.g., Öhman & Mineka, 2003) during a breaking continuous flash suppression paradigm (Jiang et al., 2007) seems to rely on low instead of high spatial frequency information (Gomes et al., 2018; for a discussion on these results see Gayet et al., 2019; Gomes et al., 2019). In sum, these results indicate that not only affective content modulates the responses to different categories of visual stimuli but also some of their low-order visual properties, which are important to control when studying affective processing. Thus, following similar procedures to the ones already employed in other stimuli sets (e.g., EmoMadrid; Carretié et al., 2019), in study 2 we also (1) provide descriptive data regarding luminosity, contrast, spatial frequency distribution, and chromatic complexity for each image, (2) conducted an exploration of these properties per emotion-related group of images, and (3) examine their associations with image valence and arousal ratings.

## Study 1

Study 1 constituted the first step in validating the colored version of OBNIS images for a Portuguese population. The normative values of the Japanese (Shirai & Watanabe, 2022) and Portuguese populations were directly compared, and their differences and similarities were discussed. Hence, for comparison purposes, the same methodology employed in the original article (see study 1 from Shirai & Watanabe, 2022) was used here.

### Method

#### Participants

In line with the original study, a total of 101 Portuguese participants (50 females), aged between 18 and 58 years (*M*_*Age*_ *=* 27.01 years, *SD =* 8.57), were recruited through an online crowdsourcing platform (Prolific Academic) to participate in study 1. All participants reported being Portuguese native speakers, having no psychiatric or neurological disorders, and no uncorrected visual impairments. Hence, no participant was excluded from the final analyses. Participants provided informed consent and were rewarded monetarily for their time (with 3.65€). The study was approved by the host institution's ethics committee (ISPA – Instituto Universitário) and was conducted following the American Psychological Association standards.

#### Stimuli

The stimuli used in this study were the colored version of the 200 images from the OBNIS database (Shirai & Watanabe, 2022). The colored and grayscale versions of the images from the OBNIS database can be downloaded on the original paper’s materials OSF website:

https://osf.io/pfrx4/?view_only=911b1be722074ad4aab87791cb8a72f5.

#### Procedure

After providing their informed consent, participants completed a sociodemographic questionnaire with personal information (e.g., age, gender) and started with the main task. The experimental procedure replicates the one used in validating the OBNIS database for the Japanese population (Shirai & Watanabe, 2022). Thus, participants were exposed to the 200 images from the original dataset in a randomized order. Their instruction was to rate each image on valence and arousal dimensions. Valence and arousal were assessed using nine-point scales ranging from 1 (*“extremely negative”* or *“low arousal”*, respectively) to 9 (*“extremely positive”* or *“high arousal”*, respectively). Additionally, participants were also instructed to categorize each image on the emotion it predominantly elicited in them, selecting one of three following descriptors: *“disgust”*, *“fear”*, or *“neither”*. Each image was presented and kept on the screen until all the responses were given. After completing the experiment, participants were debriefed and received monetary compensation. In total, the procedure took approximately 45 min to complete. The task was prepared using the PsychoPy experiment builder (version: 2022.2.4; Peirce et al., 2019) and presented to the participants on a browser window using the online data collection platform Pavlovia (https://pavlovia.org).

#### Data preparation and statistical analysis

##### Emotion categorization

The percentage of times that each image was classified into each emotion descriptor (i.e., *“fear”*, *“disgust”*, or *“neither”*) was computed. Then, a cluster analysis was conducted using the emotional classification percentages into the three classification options to inspect how the images were arranged in different groups. Shirai and Watanabe’s (2022) cluster analysis procedure was used. Firstly, the optimal number of clusters was estimated using the R package *NbClust* (Charrad et al., 2014). *NbClust* tests 30 different indices to identify the optimal number of clusters, selecting the ideal number by the majority rule (i.e., the optimal number of clusters proposed by the majority of the distinct indices was selected). Then, a cluster analysis was performed on the software JASP (JASP Team, 2022), using Euclidean distances, linkage method ‘ward.D2’ (Murtagh & Legendre, 2014), and the fixed number of clusters as obtained with the *NbClust* R package. Each cluster's mean percentage of each emotion classification option was computed. The name of each cluster was attributed based on the emotion that was most often used to classify the images within the cluster.

##### Valence and arousal ratings

A scatterplot was first used to visually inspect how valence and arousal related to each other in the Portuguese population for this particular set of visual stimuli.

Then, the relation between valence and arousal (i.e., how valence ratings predict arousal) was formally assessed using the six regression models proposed by Kuppens et al. (2013; see also Yik et al., 2022). Notably, how valence and arousal relate to each other is still a matter of debate in the affect research field (see Kuppens et al., 2013; Yik et al., 2022). As Kuppens et al. (2013) noted, at least six distinct theoretical models can be found in the literature attempting to describe their relation, which may depend, among others, on the stimulus conditions, culture, and language. For instance, in self-reported affect studies (e.g., Barrett & Russell, 1999; Yik et al., 2011), valence was frequently assumed to be independent of arousal (i.e., feeling more positive or negative does not relate to feeling more or less aroused; see model 1 from Yik et al., 2022).

Another possibility discussed in the literature concerns a linear relation between valence and arousal (see Tsai et al., 2006), which can be positive (ranging from sadness with negative valence and low arousal to excitement with positive valence and high arousal) or negative (ranging from tension with negative valence and high arousal to calmness with positive valence and low arousal) (see model 2 from Yik et al., 2022).

Interestingly, studies with visual scenes (e.g., Lang, 1994; Mattek et al., 2017) or emotion lexicons (Ćoso et al., 2019; Yao et al., 2017) have shown that arousal may be a symmetrical V-shape (also referred to as boomerang shape) function of valence. In other words, arousal is minimal at neutral valence, and its intensity increases with positive or negative valence symmetrically (i.e., negative and positive valence have the same intercept in the arousal axis and the value of their slopes is the same but with contrary signs; see model 3 from Yik et al., 2022).

However, considering the evaluative space model from Cacioppo and Berntson (1994), there is also the possibility that a V-shape relation between valence and arousal is not symmetrical, being these asymmetries informative about how the two dimensions relate in given stimuli set, culture and context. It is possible, for instance, that negative and positive valence trigger distinct overall levels of arousal (i.e., distinct intercepts) or even slopes with different magnitudes. This is precisely what models 4–6 from Yik et al. (2022) propose (originally proposed by Kuppens et al., 2013). Model 4 assumes different intercepts for negative and positive valence but similar slopes magnitude, model 5 assumes the same intercept but different slope magnitudes, and model 6 allows distinct intercepts and slope magnitudes (for a detailed explanation of these models and the respective regression formulas, see Kuppens et al., 2013; Yik et al., 2022).

For study 1, we examined which of the six described models best fits the obtained data. Following Kuppens et al. (2013) and Yik et al. (2022), the best-fitting model was selected by relying on the Bayesian information criterion (BIC) and the posterior probabilities derived from BIC (PostP; Raftery, 1995). The best-fitting model had the lowest BIC and the highest PostP. For these analyses, valence ratings were centered around the scale midpoint to examine whether negative and positive valenced stimuli present distinct relations with arousal ratings. These analyses were conducted using R (R Core Team, 2022).

Additionally, research has shown that different emotions have distinct valence and arousal levels (e.g., Alarcão & Fonseca, 2018; Barrett, 2012), which may also vary as a function of participants’ gender (Soares et al., 2015). Because valence and arousal are correlated, a two-way MANOVA (see Field, 2014) was conducted using the cluster classification and participants’ gender as factors and the valence and arousal ratings as dependent variables to inspect for possible differences between the three clusters and between females and males on mean valence and mean arousal ratings of the images. Following significant multivariate main effects of cluster classification and participants' gender, as well as a significant interaction between them, each dependent variable was examined using separate one-way ANOVAs.

Please note that although the assumptions of multivariate normality and the homogeneity of the covariance matrices (Field, 2014) were not always verified in our data, parametric MANOVA is robust to the violation of these assumptions in larger sample sizes (especially when the sample size allows the use of the central limit theorem; see Tabachnick & Fidell, 1996) and balanced designs (see Sharma, 1996). Moreover*, Pillai*’s test statistic was used because it is thought to be the most powerful method for heterogenous co-variances (Johnson, 1998). These criteria were used for all the MANOVAs reported in the manuscript.

The MANOVA was conducted using JASP analysis software (JASP Team, 2022), and all post hoc tests were performed with Bonferroni corrections.

##### Cross-cultural comparisons

Firstly, a descriptive comparison between the cluster solution found by Shirai and Watanabe (2022) and the cluster solution obtained in study 1 was performed to inspect whether both populations classify the same images with the same emotional descriptors.

Then the relationship between valence and arousal ratings was visually inspected for Shirai and Watanabe’s (2022) data through a scatterplot and then formally assessed using the regression models retrieved from Kuppens et al. (2013). The same procedure as described above for study 1’s data was used. We aimed to examine whether the different samples (i.e., Portuguese and Japanese) have distinct best-fitting models for the relation between valence and arousal for this specific stimulus set (see Yik et al., 2022). These analyses were performed using R (R Core Team, 2022).

Additionally, as observed in previous validations of other stimulus sets, distinct populations may evidence different valence and arousal ratings for the same set of images (e.g., Soares et al., 2015). Hence, a two-way MANOVA was conducted to examine whether the different populations rate the images classified in distinct clusters with different valence and arousal ratings. This two-way MANOVA employed the population (Japanese vs. Portuguese) and the cluster classification as factors and valence and arousal ratings as dependent variables. Once again, following the factors’ main effects and a statistically significant interaction, each dependent variable was examined in separate two-way ANOVAs.

The MANOVA was performed using the JASP analysis software (JASP Team, 2022), and all post hoc tests were performed with Bonferroni corrections. No gender comparisons between populations were performed because this data is unavailable from the original authors’ OSF website.

Notably, the percentage of emotion classification, mean valence and arousal (for the overall sample and also per gender), and cluster classification from study 1 are displayed in Table SM-1, available from the project web page on the Open Science Framework (OSF) platform (https://osf.io/b5zs7). All the data analysis steps and code are also available.

### Results and discussion

#### Emotion categorization

As observed in Shirai and Watanabe (2022), the optimal cluster solution for the overall sample, estimated using the method in *NbClust*, was three clusters. The cluster analysis conducted on emotion categorization (*R*^*2*^ = .89) resulted in a solution grouping 41 images in cluster 1, 55 images in cluster 2, and 104 images in cluster 3. Table 1 shows the mean percentage of each emotion classification option per image cluster (i.e., the mean percentage of times that the images constituting the cluster were classified in each of the available emotional descriptors). Considering each cluster’s most frequently attributed emotion, cluster 1 was named ‘disgust-related group’, cluster 2 was named ‘fear-related group’, and cluster 3 was named ‘neither-related group’.

**Table 1:** Mean percentage of each emotion classification option per image cluster in study1

	Disgust	Fear	Neither
Disgust-related group	65.76	8.62	25.62
Fear-related group	12.98	58.18	28.84
Neither-related group	4.10	2.67	93.23

**Fig. 1:**
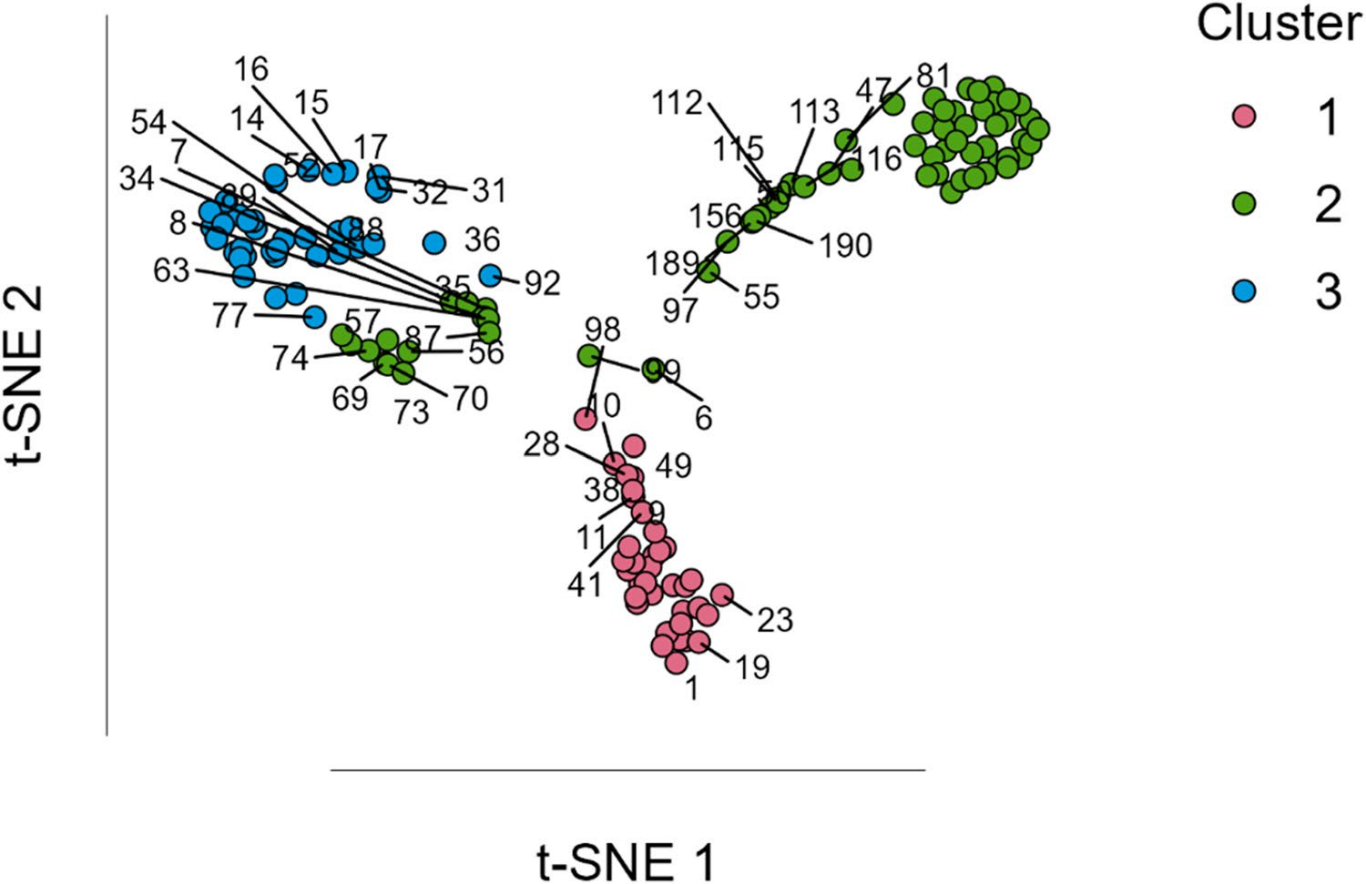
Spatial distribution of the different images in the 3 clusters using the t-SNE method (study 1). Cluster 1 is the disgust-related group, cluster 2 is the group of images associated with fear, and cluster 3 is the neither-related group. t-SNE 1 and 2 correspond to the two dimensions where the 3 clusters were represented

The cluster organization in space is represented in Fig. 1 using the t-distributed stochastic neighbor embedding (t-SNE) method, which allows the representation of high-dimensional data in a two dimensions visual map.

Notably, even though most images allocated to each cluster were mainly classified with the emotion most represented in the cluster, this was different for 16 of the 200 images. In the disgust-related group, images 6, 55, 97, and 99 were more frequently associated with the “neither” than with the “disgust” option. Regarding the fear-related group, image 92 was more frequently associated with “disgust” than with “fear”. Images 7, 8, 34, 35, 56, 63, 69, 70, 71, 73, and 87 were more frequently associated with the “neither” than with the “fear” option. For each image, the exact percentage of classification per emotional descriptor can be consulted in table SM-1 (available at: https://osf.io/b5zs7). These results indicate that these 16 images are the ones that are less consistent in the Portuguese population regarding the emotion with which they are associated.

#### Valence and arousal ratings

**Fig. 2:**
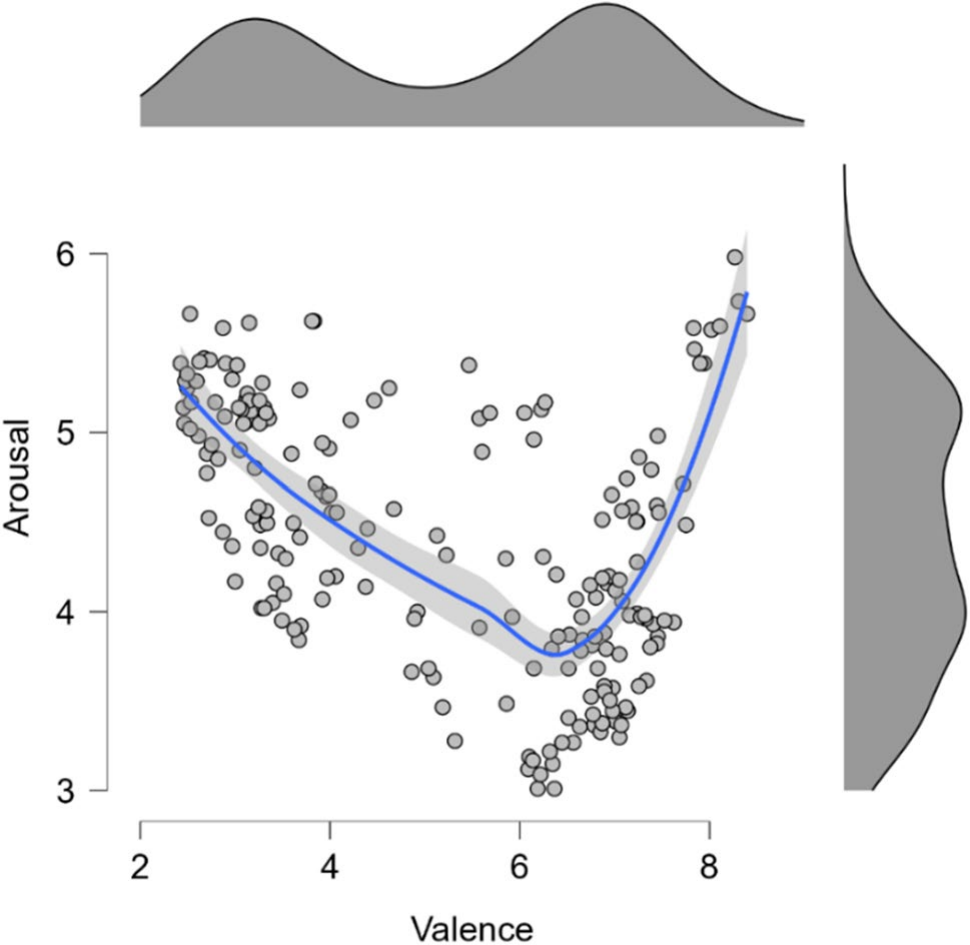
Arousal ratings represented as a function of valence ratings with a smooth regression line for the Portuguese population (study 1). Arousal was assessed using a nine-point scale ranging from 1 (“low arousal”) to 9 (“high arousal”). Valence was also assessed using a nine-point scale where 1 was associated with the label “extremely negative” and 9 with “extremely positive”. The shaded area around the regression line represents the 95% confidence interval. The graph's top and right panels represent the distribution of the valence and arousal ratings, respectively

Using a scatterplot (see Fig. 2), it is possible to observe that, as reported in previous studies (e.g., Lang, 1994; Mattek et al., 2017; Soares et al., 2015), arousal ratings seem to be a V-shape function of valence ratings. That is, arousal is minimal around the valence scale midpoint and increases for valence values closer to the extremes of the scale (either values associated with negative or positive valence ratings).

As previously mentioned in the Data preparation and statistical analysis section, the relationship between valence and arousal was formally assessed using the regression models proposed by Kuppens et al. (2013; see also Yik et al., 2022). For more information about all the examined models and the selection statistics used, see Table 2.

**Table 2:** Summary of the distinct models examined and the selection statistics when arousal is modeled as a function of valence for the Portuguese population

	Models' equations	Adjust. *R*^2^	BIC	PostP
Model 1 - Independence	Arousal_i_ = β_0_ + ε_i_	NA	446.03	.00
Model 2 - Linear relation	Arousal_i_ = β _0_ + β _1_Valence_i_ + ε_i_	.19	408.52	.00
Model 3 - Symmetric V	Arousal_i_ = β _0_ + β _1_|Valence_i_| + ε_i_	.08	432.84	.00
Model 4 - Asymmetric V with distinct intercepts	Arousal_i_ = β _0_ + β _1_|Valence_i_| + β _2_I_i_ + ε_i_	**.36**	**363.97**	**.92**
Model 5 - Asymmetric V with distinct slopes	Arousal_i_ = β _0_ + β _1_|Valence_i_| + β _3_I_i_|Valence_i_| + ε_i_	.34	372.95	.01
Model 6 - Asymmetric V with distinct intercepts and slopes	Arousal_i_ = β _0_ + β _1_|Valence_i_| + β _2_I_i_ + β _3_I_i_|Valence_i_| + ε_i_	.36	369.09	.07

In all models, the valence was centered around the scale midpoint (i.e., 5); In the models' equations, the ‘I’ denotes a dummy variable that indicates whether valence is negative (I = 0; valence rating lower than the scale midpoint) or positive (I = 1; valence rating higher than the scale midpoint), allowing testing for different intercepts and slopes for negative and positive valenced stimuli; The values of the model that best fits study's 1 data are displayed in bold. BIC = Bayesian information criterion (lower values indicate better fit); PostP = Posterior probability of each model considering the data of the six tested models

**Fig. 3:**
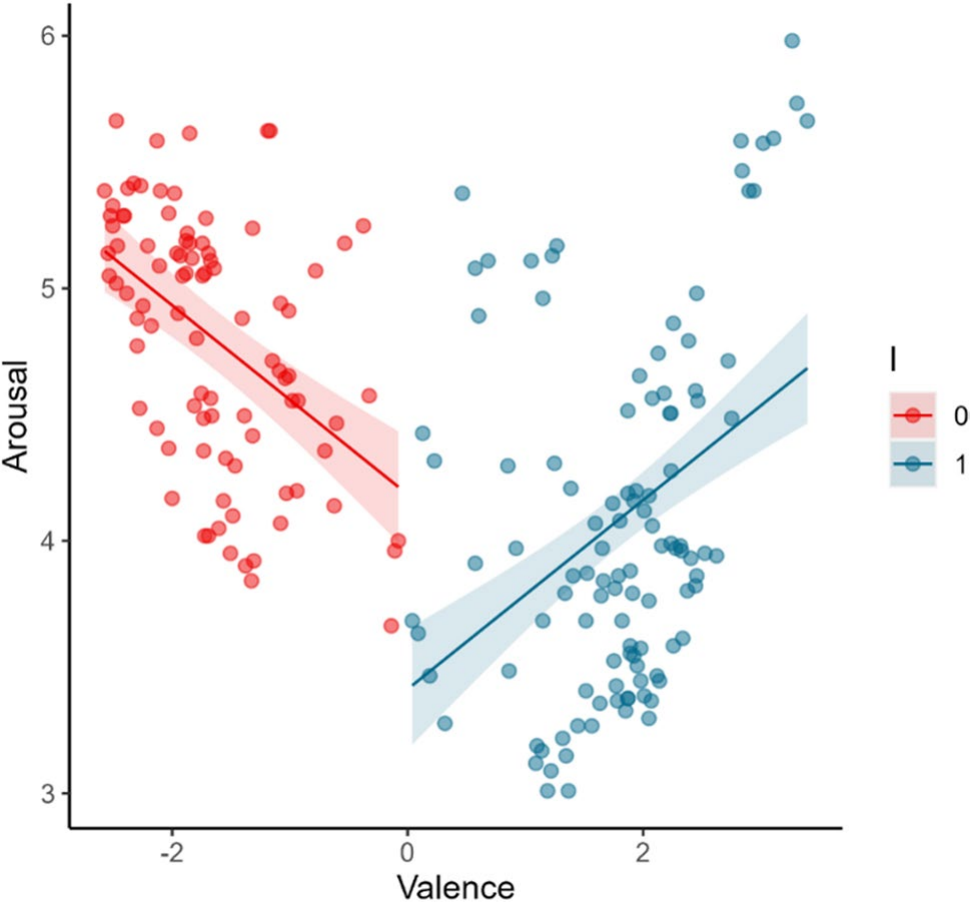
Arousal ratings represented as a function of valence ratings for the Portuguese population as predicted by model 4. The *shaded area* around the regression line represents the 95% confidence interval. Valence is centered around the scale midpoint. ‘I’ denotes a dummy variable that indicates whether valence is negative (I = 0) or positive (I = 1)

Model 4 was revealed to be the model that best fits the study’s 1 data. This regression model [*Adjust. R*^*2*^ = .36; *F*(2, 197) = 58.05, *p* < .001], which assumes an asymmetric V-shape with distinct intercepts but equal slope magnitude for negative and positive valenced stimuli, revealed that the absolute value of valence ratings (centered around the scale midpoint, i.e., 5) significantly predict the arousal ratings (*B* = .37, *t* = 6.25, *p* < .001). This supports that, for the Portuguese population, arousal is a V-shape function of valence when the OBNIS image set is considered. Additionally, a significant effect of a dummy variable (‘I’) distinguishing between negative and positive valenced stimuli (i.e., I = 0 meaning that the valence rating was lower than the scale midpoint or I = 1 indicating that the valence rating was higher than the scale midpoint) was also found (*B* = –.77, *t* = –9.40, *p* < .001). This confirms the asymmetry of the relation, indicating that the slopes for negative and positive valenced stimuli have distinct intercepts. The observed negative unstandardized beta (B) shows that the arousal slope for negative valence has a higher intercept than for positive valence, pointing to a negativity offset. In other words, the OBNIS’ negative valenced stimuli are associated with higher arousal ratings in general than the positive valenced stimuli by the Portuguese population. Moreover, model 4 was the one that best fit the data, and considering that it did not allow different slopes for negative and positive valenced stimuli (i.e., the interaction between the dummy variable ‘I’ and valence was not included in the model), it is also possible to conclude that for the Portuguese population, arousal increases similarly for negative and positive valenced stimuli (see Fig. 3).

Additionally, to examine whether valence and arousal ratings were significantly different between the distinct cluster of images and between female and male participants, a two-way MANOVA was conducted. A main effect of gender [*Pillai’s Trace* = 0.10; *F*(2, 393) = 22.30, *p* < .001], a main effect of the image cluster [*Pillai’s Trace* = 0.96; *F*(4, 788) = 182.52, *p* < .001], as well as a significant interaction between gender an image cluster [*Pillai’s Trace* = 0.07; *F*(4, 788) = 7.43, *p* < .001] were revealed.

Separate one-way ANOVAs (one for valence and another for arousal) were used to further explore each dependent variable.

Considering valence, a main effect of gender was confirmed [*F*(1, 394) = 34.98, *p* < .001, *η*_*p*_^*2*^ = .08]. Female participants (*M* = 5.03, *SD* = 2.06) rated images in general as more negative than male participants (*M* = 5.42, *SD* = 1.66). Importantly a significant interaction between participants’ gender and image cluster was also found [*F*(2, 394) = 10.21, *p* < .001, *η*_*p*_^*2*^ = .05]. Female participants rated disgust (*M* = 2.77, *SD* = 0.50) and fear (*M* = 3.39, *SD* = 1.10) images as more negative than male participants (disgust: *M* = 3.48, *SD* = 0.57, *t* = –4.27, *p*_*Bonferroni*_ < .001; fear: *M* = 4.16, *SD* = 0.99, *t* = –4.90, *p*_*Bonferroni*_ < .001). However, no significant differences were observed between gender for the neither-related group of images (females: *M* = 6.80, *SD* = 0.90; males: *M* = 6.82, *SD* = 0.65; *t* = –0.24, *p*_*Bonferroni*_ = 1.000). These results indicate that the overall lower valence ratings observed for female participants were driven by stimuli associated with the negative emotions of fear and disgust. As expected, a main effect of the image cluster was also observed [*F*(2, 394) = 809.63, *p* < .001, *η*_*p*_^*2*^ = .80]. Overall, disgust-related images (*M* = 3.16, *SD* = 0.66) were rated as significantly more negative than fear (*M* = 3.78, *SD* = 1.11; *t* = –5.14, *p*_*Bonferroni*_ < .001) and neither-related images (*M* = 6.81, *SD* = 0.79; *t* = –33.93, *p*_*Bonferroni*_ < .001). Fear-related images were also rated as significantly more negative than the neither-related ones (*t* = –31.16, *p*_*Bonferroni*_ < .001).

Concerning arousal, the ANOVA also revealed a main effect of gender [*F*(1, 394) = 6.22, *p* = .013, *η*_*p*_^*2*^ = .02]. Female participants (*M* = 4.52, *SD* = 0.69) rated images, in general, as more arousing than male participants (*M* = 4.30, *SD* = 0.79). A significant interaction between gender and image cluster was also found [*F*(2, 394) = 7.60, *p* < .001, *η*_*p*_^*2*^ = .04]. Images in the neither-related group were rated by female participants (*M* = 4.21, *SD* = 0.49) as more arousing than male participants (*M* = 3.79, *SD* = 0.63; *t* = 5.30, *p*_*Bonferroni*_ < .001). Interestingly, no significant differences were observed between female and male participants regarding the arousal ratings of the images in the disgust (females: *M* = 4.62, *SD* = 0.49; males: *M* = 4.48, *SD* = 0.46, *t* = 1.07, *p*_*Bonferroni*_ = 1.000) and fear-related (females: *M* = 5.03, *SD* = 0.29; males: *M* = 5.13, *SD* = 0.44, *t* = –0.87, *p*_*Bonferroni*_ = 1.000) groups. These results indicate that the observed overall differences between female and male participants regarding arousal ratings are related to the images in the neither-related group. As expected, a main effect of the image cluster was also revealed [*F*(2, 394) = 130.36, *p* < .001, *η*_*p*_^*2*^ = .40]. Overall, images in the fear-related group (*M* = 5.08, *SD* = 0.37) were rated as more arousing than the images in the disgust (*M* = 4.55, *SD* = 0.47; *t* = 6.36, *p*_*Bonferroni*_ < .001), and neither-related (*M* = 4.00, *SD* = 0.72; *t* = 15.96, *p*_*Bonferroni*_ < .001) groups. Moreover, images in the disgust-related group were also rated as more arousing than the neither-related ones (*t* = 7.32, *p*_*Bonferroni*_ < .001). These results are summarized in Table 3.

**Table 3:** Mean values and standard deviations (in parenthesis) of valence and arousal ratings per image cluster and participants’ gender (study 1)

		Disgust-related	Fear-related	Neither-related
Valence	Overall	3.16 (0.51)	3.78 (1.03)	6.81 (0.77)
	Female	2.77 (0.50)	3.39 (1.10)	6.80 (0.90)
	Male	3.55 (0.57)	4.16 (0.99)	6.82 (0.65)
Arousal	Overall	4.55 (0.46)	5.08 (0.33)	4.00 (0.67)
	Female	4.62 (0.49)	5.03 (0.29)	4.21 (0.74)
	Male	4.48 (0.46)	5.13 (0.44)	3.79 (0.63)

#### Cross-cultural comparisons

Regarding emotion discrimination, we compared descriptively the cluster solutions for Portuguese and Japanese populations. We found that only 12% of the images (24 images) were categorized in a different cluster. Thirteen (i.e., images 7, 8, 15, 16, 17, 31, 32, 33, 34, 35, 36, 63, and 87) of these 24 images were categorized as disgust by the Japanese sample. These were included in the fear-related group by the Portuguese sample. The remaining 11 images (47, 50, 80, 81, 112, 113, 114, 116, 189, 190, and 192) were assigned to the neither-related group in the current study (Portuguese population) (see table SM-1 in https://osf.io/b5zs7). These results indicate that, when just three emotional descriptors are considered, there is a high agreement between populations regarding the emotions elicited by the OBNIS images.

**Fig. 4:**
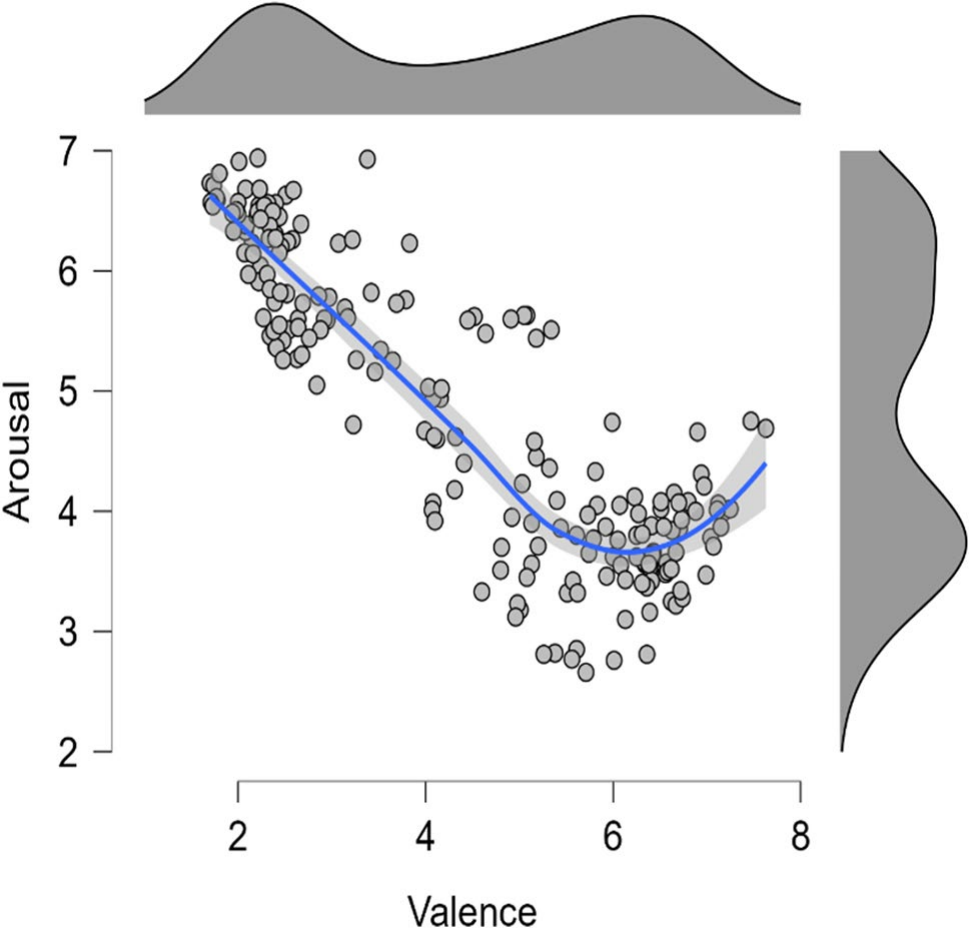
Arousal ratings represented as a function of valence ratings with a smooth regression line for the Japanese population (data from Shirai & Watanabe, 2022). Arousal was assessed using a nine-point scale ranging from 1 (“low arousal”) to 9 (“high arousal”). Valence was also assessed using a nine-point scale where 1 was associated with the label “extremely negative” and 9 with “extremely positive”. The shaded area around the regression line represents the 95% confidence interval. The graph's top and right panels represent the distribution of the valence and arousal ratings, respectively

Regarding the relation between valence and arousal for the Japanese population (data from Shirai & Watanabe, 2022), a scatter plot shows that the V-shape function obtained for the Portuguese population is not so evident in the case of the Japanese population (see Fig. 4). A visual inspection shows that the Japanese population does not appear to rate the positively valenced stimuli with higher levels of arousal as the Portuguese.

When the relation between valence and arousal ratings for the Japanese population was formally examined using the regression models proposed by Kuppens et al. (2013; see also Yik et al., 2022), it was revealed that, differently from the Portuguese population (where the model 4 was selected), model 5 was the one that best fits Japanese population data (for more information about all the examined models and the selection statistics used, see Table 4).

**Table 4:** Summary of the distinct models examined and the selection statistics when arousal is modeled as a function of valence for the Japanese population

	Models' equations	Adjust. R^2^	BIC	PostP
Model 1 - Independence	Arousal_i_ = β_0_ + ε_i_	NA	658.37	.00
Model 2 - Linear relation	Arousal_i_ = β _0_ + β _1_Valence_i_ + ε_i_	.75	385.97	.00
Model 3 - Symmetric V	Arousal_i_ = β _0_ + β _1_|Valence_i_| + ε_i_	.48	533.35	.00
Model 4 - Asymmetric V with distinct intercepts	Arousal_i_ = β _0_ + β _1_|Valence_i_| + β _2_I_i_ + ε_i_	.74	400.28	.00
Model 5 - Asymmetric V with distinct slopes	Arousal_i_ = β _0_ + β _1_|Valence_i_| + β _3_I_i_|Valence_i_| + ε_i_	**.80**	**350.33**	**.84**
Model 6 - Asymmetric V with distinct intercepts and slopes	Arousal_i_ = β _0_ + β _1_|Valence_i_| + β _2_I_i_ + β _3_I_i_|Valence_i_| + ε_i_	.80	353.67	.16

In all models, the valence was centered around the scale midpoint (i.e., 5); In the models' equations, the I denotes a dummy variable that indicates whether valence is negative (I= 0; valence rating lower than the scale midpoint) or positive (I= 1; valence rating higher than the scale midpoint), allowing testing for different intercepts and slopes for negative and positive valenced stimuli; The values of the model that best fit Japanese population’s data are displayed in bold. BIC = Bayesian information criterion (lower values indicate better fit); PostP = Posterior probability of each model considering the data of the six tested models

**Fig. 5:**
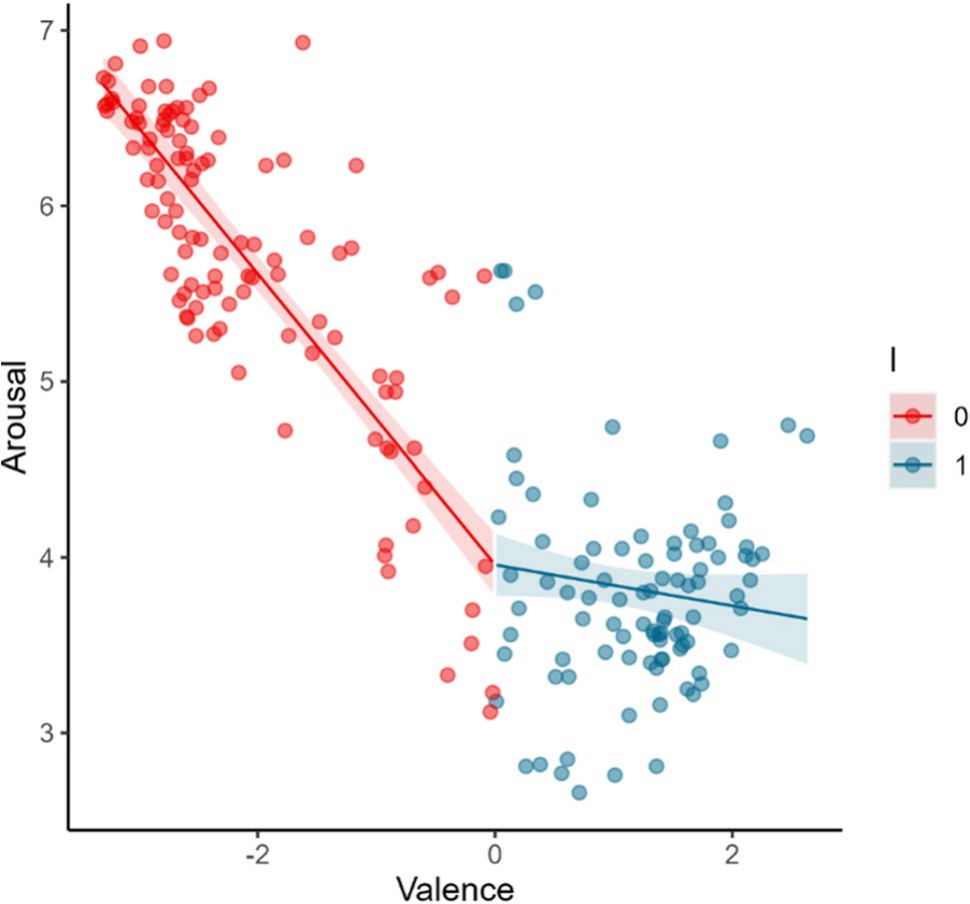
Arousal ratings are represented as a function of valence ratings for the Japanese population as predicted by model 5. The shaded area around the regression line represents the 95% confidence interval. Valenced is centered around the scale midpoint. ‘I’ denotes a dummy variable that indicates whether valence is negative (I= 0) or positive (I= 1)

This model assumes arousal as an asymmetric function of valence with negative and positive slopes having the same intercept in the arousal axis but different magnitudes (i.e., model 5 includes the interaction between valence and ‘I’, a dummy variable distinguishing between negative and positive valenced stimuli, but not the main effect of ‘I’). The regression model [*Adjust*. *R*^*2*^= .80; *F*(2, 197) = 386.05, *p* < .001], revealed not only that the absolute value of valence ratings (centered around the scale midpoint, i.e., 5) significantly predict arousal ratings (*B* = .83, t = 19.34, *p* < .001), but also shows a significant interaction between valence and the dummy variable ‘I’ (*B* = –.95, t = –17.55, *p* < .001). Analyzing the unstandardized betas (B) it is possible to conclude that the slope magnitude for negatively valenced stimuli is much higher than for positively valenced stimuli, with the latter being almost flat (see Fig. 5).

These results indicate that for the Japanese population, the more negative a stimulus is, the more arousal it elicits (a trend also observed in study’s 1 data). However, contrary to the Portuguese population, the same is not true for positively valenced stimuli, with arousal not substantially increasing with an increase in valence.

**Fig. 6:**
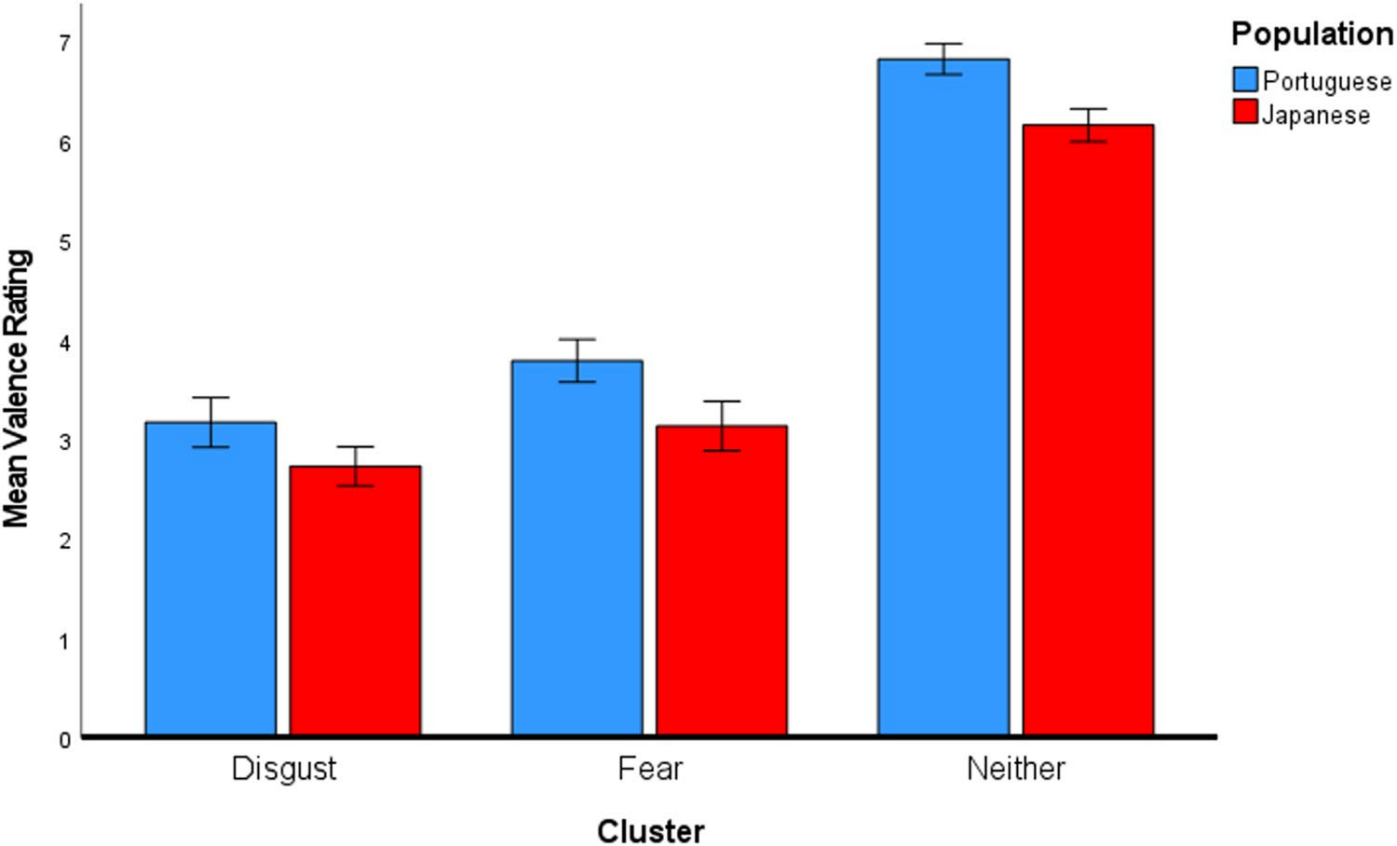
Mean valence for each image cluster per population. *Error bars* represent the 95% confidence intervals

Moreover, we examined possible differences between the two populations regarding valence and arousal ratings using a two-way MANOVA. The aim was to inspect whether the two populations rated the images classified in distinct clusters with different valence and arousal ratings. This MANOVA employed the population (Portuguese or Japanese) and cluster classification as factors and valence and arousal ratings as dependent variables. A main effect of the population [*Pillai’s Trace* = 0.27; *F*(2, 393) = 72.11, *p* < .001], a main effect of the cluster [*Pillai’s Trace* = 0.95; *F*(4, 788) = 177.52, *p* < .001], as well as a significant interaction between the population and cluster, were revealed [*Pillai’s Trace* = 0.29; *F*(4, 788) = 32.67 *p* < .001]. Hence, two separate ANOVAs (one for valence and another for arousal) were conducted. Regarding valence, a main effect of the population [*F* (1, 394) = 46.14, *p* < .001, *η*_*p*_^*2*^ = .11] was confirmed. Japanese participants (*M* = 4.40, *SD* = 1.83) reported lower valence ratings than Portuguese participants (*M* = 5.23, *SD* = 1.85). Not surprisingly, a main effect of the cluster was also observed [*F* (2, 394) = 819.61, *p* < .001, *η*_*p*_^*2*^ = .81]. Regardless of the population, images in the disgust-related group (*M* = 2.89, *SD* = 0.74) were rated as significantly more negative than the images in the fear (*M* = 3.50, *SD* = 1.08; *t* = –4.41, *p*_*Bonferroni*_ < .001), and neither-related (*M* = 6.50, *SD* = 0.80; *t* = –35.73, *p*_*Bonferroni*_ < .001) groups. Images in the fear-related group were also rated as more negative than images in the neither-related group [*t*= –29.82, *p*_*Bonferroni*_ < .001]. Lastly, no significant interaction between population and cluster was revealed [*F* (2, 394) = 0.71, *p* = .494, *η*_*p*_^*2*^ = .00]. These results indicate that, although lower for the Japanese population, valence ratings follow the same trend in both populations for the different image clusters (see Fig. 6).

**Fig. 7:**
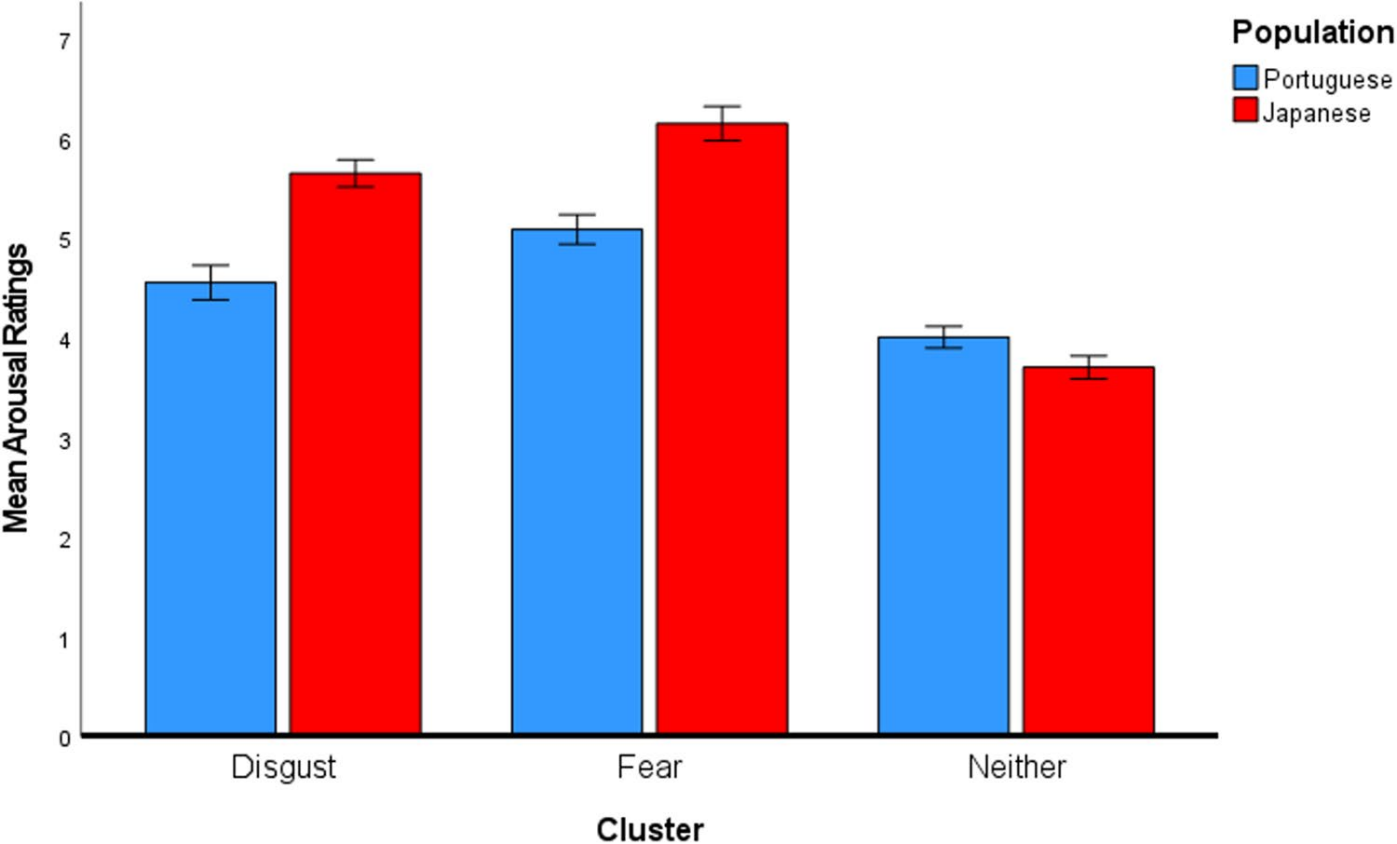
Mean arousal for each image cluster per population. Error bars represent the 95% confidence intervals

In what concerns the arousal ratings, the ANOVA also revealed a main effect of the population [*F* (1, 394) = 106.54, *p* < .001, *η*_*p*_^*2*^ = .21], with Japanese participants (*M* = 4.84, *SD* = 1.23) reporting in general higher arousal levels than Portuguese participants (*M* = 4.41, *SD* = 0.72). Importantly a population and cluster interaction was found [*F* (2, 394) = 74.17, *p* < .001, *η*_*p*_^*2*^ = .27]. The Japanese population rated images in the disgust-related group (*M* = 5.65, *SD* = 0.74) as more arousing than the Portuguese population (*M* = 4.55, *SD* = 0.46; *t* = 9.81, *p*_*Bonferroni*_ < .001). The same is true for the images in the fear-related group, with the Japanese population (*M* = 6.15, *SD* = 0.49) rating them as more arousing than the Portuguese population (*M* = 5.08, *SD* = 0.33; *t* = 9.18, *p*_*Bonferroni*_ < .001). Congruently with what was observed in the regression models mentioned earlier, the data pattern is reversed when the neither-related group is considered. The Portuguese population rated the images in the neither-related group (*M* = 4.00, *SD* = 0.67) as significantly more arousing than the Japanese population (*M* = 3.70, *SD* = 0.46; *t* = 3.78, *p*_*Bonferroni*_ = .003), indicating that the Portuguese population attributed higher arousal ratings to stimuli not associated with the negative emotions. Not surprisingly, a main effect of the cluster [*F* (2, 394) = 368.11, *p* < .001, *η*_*p*_^*2*^ = .65] was also found for arousal ratings. Overall, the images in the fear-related group (*M* = 5.38, *SD* = 0.66) were rated as significantly more arousing than the images in the disgust (*M* = 5.23, *SD* = 0.84; *t* = 6.43, *p*_*Bonferroni*_ < .001) and neither-related (*M* = 3.86, *SD* = 0.60; *t* = 25.05, *p*_*Bonferroni*_ < .001) groups. Images in the disgust-related group were also rated as more arousing than the images in the neither-related group (*t* = 18.15, *p*_*Bonferroni*_ < .001) (see Fig. 7).

In sum, Japanese participants generally assessed the OBNIS images as less positive. Furthermore, the images in the disgust and fear-related groups (i.e., mostly the negatively valenced ones) were judged by the Japanese as more arousing than Portuguese participants. Regarding the images in the neither-related group (i.e., mostly the positively valenced ones), the Portuguese found them more arousing than the Japanese participants. These results indicate that the Portuguese population may associate some images in the neither-related group with positive emotions (e.g., happiness). This is an aspect that was examined in study 2.

## Study 2

As observed in study 1, Japanese and Portuguese populations present remarkable differences in their affective reactions to the OBNIS stimuli, especially when the arousal ratings of positively valenced stimuli are considered. Hence, the first aim of study 2 was to examine whether the Portuguese population associates some of the OBNIS stimuli with emotions other than fear and disgust (e.g., happiness). To achieve our goal, in study 2, instead of the 3 classification options (disgust, fear, or neither), we used six emotional descriptors (fear, disgust, sadness, surprise, anger, happiness; i.e., the six basic emotions defined by Ekman, 1992) plus a “neither” option.

Additionally, study 2 was conducted in a laboratory context, with the stimuli visualization conditions being constant across participants (i.e., equal screens, same image display definitions, and similar view distances). This enabled the exploration of the relation of stimuli’s low-order visual properties (i.e., luminosity, contrast, spatial frequency distribution, and chromatic complexity) with emotional classification and arousal and valence ratings. Descriptive data per image of each of those mentioned above low-order visual properties is also provided.

### Method

#### Participants

A total of 208 university students (177 female) were recruited using the host institution’s participants pool, gave their informed consent, and participated in the experiment voluntarily. Thirteen participants were excluded, three for disclosing they were color-blind, and ten for not being Portuguese. Hence, 195 participants aged 18–59 years (167 females; *M*_*Age*_ *=* 22.53 years; *SD =* 6.94) were included in the final analysis. All participants were psychology students who reported no psychiatric or neurological disorders and no uncorrected visual impairments. Participants were rewarded for their participation with course credits. The study was approved by the host institution's ethics committee (ISPA – Instituto Universitário) and was conducted following American Psychological Association standards and the guidelines of the Declaration of Helsinki. The experimental procedure was preregistered in Open Science Framework (OSF): https://osf.io/75evj.

#### Stimuli

The 200 images from the OBNIS database (Shirai & Watanabe, 2022), in their colored version, constituted the stimuli used in this study. The stimuli were then scaled from their original size (500 by 500) to an area of 300 by 300 pixels using Adobe Photoshop CS6 (Adobe Inc, 2012). This size reduction was performed to make the images better fit the display conditions on the laboratory computer screens. The images were scaled previously to the presentation (instead of resizing them through the presentation platform) to ensure that the low-order properties were calculated considering the specific image presentation parameters. It is important to note that the quality of the images was not affected, just their size.

As already mentioned in study 1, the image set created initially by Shirai and Watanabe (2022), i.e., the 500 by 500 px version of the images, and the images’ grayscale version, can be found and freely downloaded on the authors’ OSF website: https://osf.io/pfrx4/?view_only=911b1be722074ad4aab87791cb8a72f5.

The colored images resized to an area of 300 by 300 pixels used in study 2 can be found at: https://osf.io/b5zs7.

#### Low-order visual properties

Such as in other affective image sets (e.g., EmoMadrid; Carretié et al., 2019), several low-order visual descriptors, potentially critical in affective sciences, were computed for each image (i.e., luminosity, contrast, chromatic complexity, spatial frequency distribution) using MATLAB (R2017b). The aim was to characterize the data set as it is made available to the community. In this regard, and in light of some varying properties among stimuli, first, the area ratio was computed, expressing the area of the whole image occupied by the stimuli (i.e., excluding the background). Considering the differences in the area among stimuli (*M*_*ratio*_= .28, *SD* = .12, range = .06–.66), the mean luminance and root-mean-square (RMS) contrast properties were computed solely for the stimuli, excluding the background, since computing the whole image properties would be biased by different stimuli sizes.

Effective contrast was computed for the whole image. Since this measure is dependent on the dimension of the stimuli, when presented to a viewer, we considered common properties of an experimental setting with the viewer positioned at 50 cm from the screen and the stimuli occupying circa 4° of the visual angle.

Such as, in Carretié et al. (2019), chromatic complexity was computed using the file size in kilobytes of each image in JPEG format. The file size is proportional to color changes between neighbor pixels in this file format. Hence, image areas with the same color are codified with less information than areas including several color changes. In such a way, JPEG images with larger file sizes have greater chromatic complexity than images with smaller files (see Marchewka et al., 2014; Müller et al., 2008).

SF concerns how frequently a property of the image change in space. Hence, stimuli with fine detail (e.g., sharp edges) have more energy in high spatial frequencies (HSF; i.e., more variations in space), while stimuli with fewer variations (e.g., stimuli without fine details, such as a monochromatic circle) have more energy in lower frequencies (LSF) (see Delplanque et al., 2007). It is important to note that, for a given picture, the perceived content by the observers also depends on the view distance (Sowden & Schyns, 2006). The higher the distance, the lower the HSF (i.e., fine details) perceived components. Thus, when one is interested in studying the association between SF and affective processing, it is important to keep the distance between the picture and observer approximately constant. For the present research, SF was computed using the procedures and the MATLAB script provided by Delplanque et al. (2007). Specifically, we computed the spectral energies in pixels per degree (p/c) for nine frequency bands (300–150 p/c, 150–75 p/c, 75–38 p/c, 38–19 p/c, 19–9 p/c, 9–5 p/c, 5–2 p/c, 2–1 p/c, and residuals) for the grayscale (709 option) layer of each picture (Delplanque et al., 2007; see also Vimal, 2002).

#### Procedure

After providing their informed consent, participants were asked to complete a sociodemographic questionnaire with some personal information (e.g., age and gender) followed by the main task. Contrary to study 1, participants in study 2 did not assess all 200 images. Instead, they were presented with a random selection of just 50 images from the OBNIS. As described below, in study 2, participants had more emotional descriptors and one additional rating (emotion intensity) per image. Reducing the number of images presented to each participant allowed keeping data collection sessions shorter than 30 min, reducing potential fatigue effects. Images presentation was performed on a browser window using the Qualtrics platform (Qualtrics, Provo, UT, USA).

Participants were instructed to classify each image to one of the six basic emotions (i.e., fear, disgust, sadness, surprise, anger, happiness) or neither, according to what they predominantly felt when exposed to it. Additionally, they were instructed to rate each image on three dimensions – emotional intensity, valence, and arousal.

Emotional intensity (Strauss & Allen, 2008) was an extra rating dimension introduced in study 2, which is also referred to in the literature as emotionality (John, 1988) or emotional magnitude (Holmes & Lourenco, 2011). Some authors consider this as another dimension of emotion (e.g., Pitt & Casasanto, 2016) that refers to the extent that a given stimulus conveys emotional content. The emotional intensity was assessed here using a 100-point slider, ranging from 0 (very weak) to 100 (very strong). Participants were asked to rate how intensely they felt the emotion they selected in the classification phase. Importantly, this resulted in ratings of emotional intensity for several emotional descriptors for each image. Moreover, the emotional intensity ratings per emotional descriptor were computed from a number of data points that vary considerably among images and selected emotional descriptors, compromising the reliability of this measure in the current study. Thus, this variable was not further explored in the conducted statistical analysis. The emotional intensity was just reported in the supplementary materials for descriptive purposes only (see SM-2 at: https://osf.io/b5zs7).

Valence was rated on a 100-point slider, with 0 corresponding to an extremely negative image and 100 to an extremely positive one. Arousal was also rated with a 100-point slider ranging from 0 (low arousal) to 100 (high arousal). Notably, instead of the Likert scales used in study 1 (and also in Shirai & Watanabe, 2022), continuous 0–100 sliders were used in study 2. This methodological change was employed because sliders constitute a more sensitive measurement, providing the advantage of collecting continuous data, which eases the use of diverse parametrical statistical tests and increases the power of goodness of fit tests (see Funke & Reips, 2012; Matejka et al., 2016).

Each image was presented on the screen until all the responses were obtained. The stimuli were displayed on Asus VX238H 23” Full HD LED monitors (1920×1080) with a screen refresh rate of 60 Hz, connected to computers Dell OptiPlex 755. Participants’ chairs were placed to maintain a visual distance of approximately 55 cm. The size of the stimuli was 8 per 8 visual degrees (i.e., 300 per 300 px).

At the end of the experimental procedure, participants were debriefed and received course credit as compensation for their participation. The experimental procedure took approximately 25 min.

Notably, the experiment was prepared to achieve a minimum of 50 independent assessments per image. However, after excluding the participants who did not respond to the inclusion criteria, we obtained a mean of 48.75 data points per image (*SD* = 1.60; range, 43–52)^1^.

#### Data preparation and statistical analysis

##### Emotion categorization

Regarding the emotion classification, similar to study 1, we first computed the percentage of times each image was classified in each of the seven response options (i.e., fear, disgust, sadness, surprise, anger, happiness, or neither). Then, a cluster analysis, as described in study 1, was conducted with the 200 images for the seven classification percentages to examine how the different images are classified into distinct groups. As in study 1, the best clustering schema was found using the R package *NbClust* (Charrad et al., 2014). Then, the cluster analysis was performed in the software JASP (JASP Team, 2022), as described in study 1. The cluster solution presented in study 2 was then descriptively compared with the solution presented in study 1.

##### Valence and arousal ratings

Firstly, the relation between arousal and valence ratings was visually inspected using a scatterplot. Then, bivariate Pearson correlations were performed to examine whether the valence and arousal ratings obtained in study 2 correlate with the ones observed in study 1. These correlations were used to assess whether these two ratings can be compared across studies despite the methodological changes introduced in study 2. Please note that no further analyses were conducted as the goal of study 2 was not to examine the relationship between valence and arousal ratings for OBNIS images in the Portuguese population.

Following a similar procedure as in study 1, the possible differences between clusters regarding the mean valence and arousal ratings of the images were examined using a one-way MANOVA. The cluster classification was employed as a factor and the valence and arousal ratings as dependent variables. Following a significant multivariate main effect of the cluster classification, each dependent variable was examined using separate one-way ANOVAs.

Notably, no gender comparisons were made in study 2 because the participants' sample was not gender-balanced.

##### Low-order visual properties

Concerning luminosity, RMS contrast, and effective contrast, possible differences between the images constituting the distinct clusters were inspected using a one-way MANOVA employing cluster classification as a factor and several low-order visual properties as dependent variables. Once again, following a significant multivariate main effect of the cluster classification, each of the dependent variables was examined using separate one-way ANOVAs.

Moreover, another one-way MANOVA employing cluster classification as a factor and the nine SF bands as dependent variables was used to examine possible differences in SF distribution between clusters. Following a significant main effect of the cluster, as in Carretié et al. (2019), nine separate one-way ANOVAs (one per frequency band plus residuals) were conducted employing the cluster as the between-subjects factor. Lastly, bivariate Pearson correlations were used to inspect whether the low-order visual properties correlated with valence and arousal ratings.

All post hoc comparisons were performed using the Bonferroni correction procedure. All the analyses were performed using the software JASP (JASP Team, 2022).

### Results

#### Emotion categorization

**Fig. 8:**
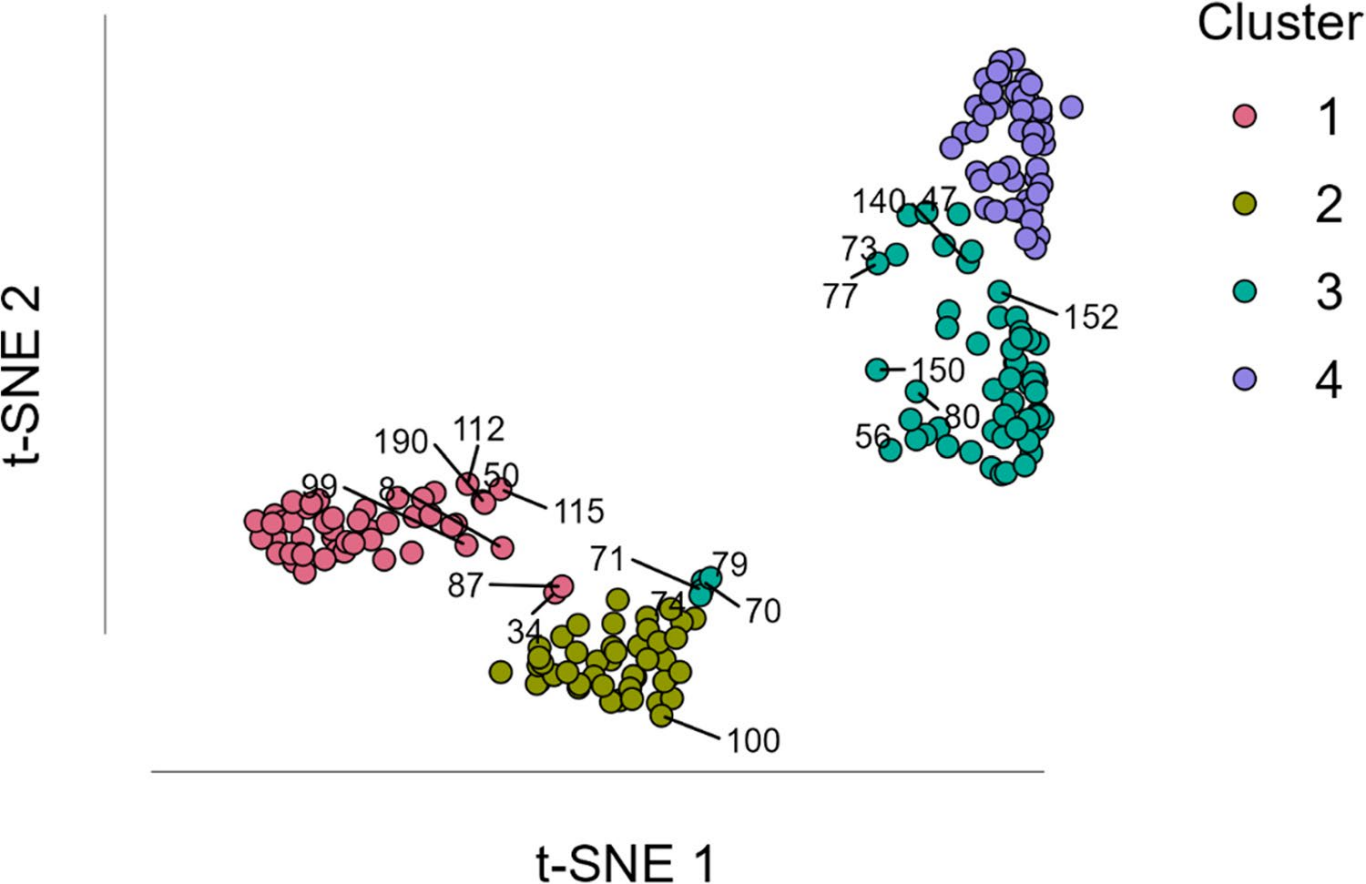
Spatial distribution of the different images in the four clusters using the t-SNE method. Cluster 1 is the disgust-related group, cluster 2 is the fear group, cluster 3 constitutes the images not associated with any emotion (the neither-related group), and cluster 4 constitutes the group with happiness-related images. t-SNE 1 and 2 correspond to the two dimensions representing the four clusters

Regarding the emotion categorization in study 2, the method implemented with *NbClust* indicates that a solution with four clusters would be optimal. Thus, the cluster analysis (*R*^*2*^ = .80) grouped 51 images in cluster 1, 44 images in cluster 2, 58 images in cluster 3, and 47 images in cluster 4. Table 5 shows the mean percentage of each emotion classification option per image cluster (i.e., the mean percentage of times that the images constituting the cluster were classified in each of the available emotional descriptors). Considering each cluster’s most frequent emotion, cluster 1 was labeled the ‘disgust-related group’, cluster 2 was labeled the ‘fear-related group’, cluster 3 was labeled the ‘neither-related group’, and cluster 4 constituted the ‘happiness-related group’. Figure 8 represents the clusters organized in space using the t-SNE method.

**Table 5:** Mean percentage of each emotion classification option per image cluster in study 2

	Anger	Disgust	Sadness	Fear	Surprise	Happiness	Neither
Disgust-related group	1.73	**62.39**	0.20	9.06	7.71	3.78	15.12
Fear-related group	0.68	18.73	0.36	**53.36**	12.23	4.18	10.20
Neither-related group	0.34	4.00	0.86	4.31	10.43	29.98	**49.91**
Happiness-related group	0.13	1.60	1.21	1.40	4.43	**73.23**	17.85

Values in bold are the most representative emotion in each image cluster

As in study 1, it is important to note that, although some images are classified in a given cluster, their most frequent associated emotion is not the most common one in their cluster. In study 2, this is the case for 19 of the 200 images. Considering the disgust-related group: image 8 was more associated with surprise; images 34 and 87 were more associated with fear; and images 112 and 115 were frequently more associated with the ‘neither’ option. In what concerns the fear-related group, image 17 was more associated with disgust. Regarding the neither-related group; images 47, 73, 77, 137, 140, 141, 143, 152, and 192 were more frequently associated with happiness; and images 70, 71, 74, and 79 were more frequently associated with fear. In the case of the happiness-related group, none of these inconsistencies were found. For each image, the exact percentage of classification per emotional descriptor can be consulted in table SM-2 (available at: https://osf.io/b5zs7).

Furthermore, a direct comparison between the cluster solutions found in studies 1 and 2 was also performed. We found 17 inconsistencies between the studies. Ten images that were classified in fear-related (four images: 8, 34, 36, and 87) and neither-related (six images: 50, 112, 115, 156, 189, 190) groups in study 1, were now classified in the disgust-related group. Additionally, 7 images (56, 70, 71, 73, 74, 77, and 79) that were included in the fear-related group in study 1, were now classified in the neither-related group. Importantly, all the images included in the happiness-related group in study 2 were classified in the neither-related group in study 1. These results confirm that the Portuguese population associates some of the images in OBNIS with a positive emotion (i.e., happiness).

#### Valence and arousal ratings

Firstly, correlation analyses were used to examine whether valence and arousal ratings obtained in study 2 resemble the ones obtained in study 1. Results indicate that both valence [*r*(198) = .98, *p* < .001] and arousal [*r*(198) = .88, *p* < .001] are highly correlated between the two studies despite all the introduced methodological changes and the sample in study 2 that was not gender-balanced. Thus, for the sake of simplicity, no further examination of the relation between valence and arousal was repeated in study 2.

A one-way MANOVA was then conducted to examine whether valence and arousal ratings (i.e., dependent variables) vary between the distinct clusters (i.e., factor). A main effect of the cluster was observed [*Pillai’s Trace* = 1.31; *F*(6, 392) = 124.94, *p* < .001]. Hence, separate ANOVAs for valence and arousal were conducted. Regarding valence, the one-way ANOVA revealed an expected main effect of the cluster [*F* (3, 196) = 444.84, *p* < .001, *η*_*p*_^*2*^ = .87]. More specifically, the images in the disgust-related group (*M* = 27.93, *SD* = 8.70) were rated as significantly more negative than the images in the neither (*M* = 55.96, *SD* = 5.33; *t* = –20.62, *p*_*Bonferroni*_ < .001) and the happiness-related (*M* = 71.15, *SD* = 6.22; *t* = –30.19, *p*_*Bonferroni*_ < .001) groups. The images in the fear-related group (*M* = 27.67, *SD* = 7.84) were also rated as significantly more negative than the images in the neither (*t* = –19.98, *p*_*Bonferroni*_ < .001) and the happiness-related (*t* = –29.27, *p*_*Bonferroni*_ < .001) groups. Moreover, images in the happiness-related group were rated as significantly more positive than the ones in the neither-related group (*t* = 10.93, *p*_*Bonferroni*_ < .001). No differences were evidenced between the disgust and fear-related groups [*t* = 0.18, *p*_*Bonferroni*_ = 1.000] (see Table 7). Concerning the arousal ratings, another main effect of the cluster was revealed [*F* (3, 196) = 58.42, *p* < .001, *η*_*p*_^*2*^ = .47]. Concretely, the images in the disgust-related group (*M* = 45.58, *SD* = 7.08) were rated as significantly less arousing than the images in the fear-related group (*M* = 54.96, *SD* = 6.47; *t* = –5.44, *p*_*Bonferroni*_ < .001) and as significantly more arousing than the images in the neither-related group (*M* = 33.95, *SD* = 9.83; *t* = 7.23, *p*_*Bonferroni*_ < .001). No significant differences were found between the disgust and happiness-related groups (*M* = 49.26, *SD* = 9.29; *t* = –2.17, *p*_*Bonferroni*_ = .187). Moreover, the images in the fear-related group were rated as significantly more arousing than the images in the neither (*t* = 12.54, *p*_*Bonferroni*_ < .001) and happiness-related groups (*t* = 3.24, *p*_*Bonferroni*_ = .008). Lastly, the images in the neither-related group were rated as less arousing than the images in the happiness-related group (*t* = –9.31, *p*_*Bonferroni*_ < .001). Table 6 summarizes these results.

**Table 6:** Mean values and standard deviations (in parenthesis) of valence and arousal ratings per image cluster (study 2)

	Disgust	Fear	Neither	Happiness
Valence	27.93 (8.70)^bc***^	27.67 (7.84)^bc***^	55.96 (5.33)^c***^	71.15 (6.22)
Arousal	45.58 (7.08)^ab***^	54.96 (6.47)^b***c**^	33.95 (9.83)^c***^	49.26 (9.29)

^a^Significant difference with the fear-related group

^b^Significant difference with the neither-related group

^c^Significant difference with the happiness-related group

** *p* < .01; *** *p* < .001

Table SM-2, available from the project web page on the Open Science Framework platform (https://osf.io/b5zs7), shows the percentage of classification in each of the emotion options, the mean intensity of the most represented emotion in the cluster in which the image is included, the mean valence and arousal, and the cluster membership of each image in study 2.

#### Low-order visual properties

##### Luminosity, contrast, and chromatic complexity

Regarding low-order visual properties, a one-way MANOVA was first conducted to examine the multivariate effects of luminosity, effective and RMS contrast, and chromatic complexity on each cluster. Following a main effect of the cluster [*Pillai’s Trace* = 0.42; *F*(12, 585) = 7.96, *p* < .001] showing differences in those low-level features between the clusters, separate ANOVAs were run for luminosity, effective and RMS contrast, and chromatic complexity.

Regarding luminosity, a main effect of the cluster was revealed [*F* (3, 196) = 19.57, *p* < .001, *η*_*p*_^*2*^ = .23]. Specifically, the images in the disgust-related group present significantly lower luminosity (*M* = 99.08, *SD* = 23.35) than the images in the neither [*M* = 123.23, *SD* = 32.54; *t* = –4.03, *p*_*Bonferroni*_ < .001] and the happiness-related groups [*M* = 140.44, *SD* = 37.07; *t* = –6.55, *p*_*Bonferroni*_ < .001]. The same was found for images in the fear-related group (*M* = 99.76, *SD* = 30.69), which showed also significantly lower luminosity when compared with the images in the neither (*t* = –3.76, *p*_*Bonferroni*_ = .001) and the happiness-related (*t* = –6.21, *p*_*Bonferroni*_ < .001) groups. In turn, the images in the happiness-related group present a significantly higher luminosity when compared with the images in the neither-related group (*t* = 2.81, *p*_*Bonferroni*_ = .033). No significant differences were evidenced between the images in the fear and the disgust-related groups (*t* = –0.11, *p*_*Bonferroni*_ = 1.000).

Concerning the RMS contrast, another one-way ANOVA also revealed a main effect of the cluster [*F* (3, 196) = 3.07, *p* = .029, *η*_*p*_^*2*^ = .05]. The only significant differences were between the disgust-related group (*M* = 9.82, *SD* = 0.78) and the happiness-related group (*M* = 10.38, *SD* = 1.03; *t* = 3.00, *p*_*Bonferroni*_ = .018), with the latter presenting a high mean RMS contrast. No statistically significant differences were found between the disgust-related group, the fear (*M* = 10.02, *SD* = 0.72; *t* = –1.06, *p*_*Bonferroni =*_ 1.000) and the neither-related (*M* = 10.04, *SD* = 1.08; *t* = –1.22, *p*_*Bonferroni*_ = 1.000) groups. No significant differences were also observed between the fear, the neither (*t* = –0.08, *p*_*Bonferroni*_ = 1.000) and happiness-related (*t* = –1.85, *p*_*Bonferroni*_ = .394) groups. Also, no differences were found between the happiness-related and the neither-related groups (*t* = 1.90, *p*_*Bonferroni*_ = .355).

The differences between the distinct clusters regarding effective contrast were also examined using a one-way ANOVA, which also revealed a main effect of the cluster [*F*(3, 196) = 3.88, *p* = .010, *η*_*p*_^*2*^ = .06]. Post hoc comparisons revealed that the images in the disgust-related group (*M* = 1.56*10^8^, *SD* = 3.98*10^7^) present a significatively lower contrast than the images in the fear (*M* = 1.79*10^8^, *SD* = 4.89*10^7^; *t* = –2.77, *p*_*Bonferroni*_ = .037) and happiness-related groups (*M* = 1.82*10^8^, *SD* = 3.66*10^7^; *t* = –3.06, *p*_*Bonferroni*_ = .015). No differences were observed between the images in the disgust and the neither-related groups(*M* = 1.73*10^8^, *SD* = 4.25*10^7^; *t* = –2.14, *p*_*Bonferroni*_ = .202). The images in the fear-related group do not present significant effective contrast differences when compared with the images in the neither (*t* = 0.79, *p*_*Bonferroni*_ = 1.000) and happiness-related (*t* = –0.23, *p*_*Bonferroni*_ = 1.000) groups. Additionally, no differences were also observed between the images in the neither and the happiness-related groups (*t* = –1.06, *p*_*Bonferroni*_ = 1.000).

Regarding the chromatic complexity, another main effect of the cluster was found [*F* (3, 196) = 9.62, *p* < .001, *η*_*p*_^*2*^ = .13]. The images in the disgust-related group (*M* = 60.84, *SD* = 20.76) show less chromatic complexity than images in the fear group(*M* = 76.55, *SD* = 27.26; *t* = –3.26, *p*_*Bonferroni*_ = .008), the neither group (*M* = 83.19, *SD* = 26.67; *t* = –4.98, *p*_*Bonferroni*_ < .001), and the happiness-related (*M* = 80.89, *SD* = 16.96; *t* = –4.24, *p*_*Bonferroni*_< .001) group. No significant differences were obtained between the images in the fear-related group and the images in the neither (*t* = –1.42, *p*_*Bonferroni*_ = .942) and happiness-related groups (*t* = 0.89, *p*_*Bonferroni*_ = 1.000). The images in the neither-related group did not differ also from the images in the happiness-related group (*t* = 0.50, *p*_*Bonferroni*_ = 1.000).

##### SF distribution

Another one-way MANOVA was conducted employing the 8-frequency band plus residuals as dependent variables and the cluster classification as factor. Following a main effect of the cluster [*Pillai’s Trace* = 0.61; *F*(27, 570) = 5.40, *p* < .001] separate one-way ANOVAs were conducted for each dependent variable. ANOVAs revealed statically significant differences between clusters for all of the examined frequency bands [300–150 p/c: *F* (3, 196) = 4.70, *p* = .003, *η*_*p*_^*2*^ = .07; 150–75 p/c: *F*(3, 196) = 3.16, *p* = .026, *η*_*p*_^*2*^ = .05; 75–38 p/c: *F*(3, 196) = 6.81, *p* < .001, *η*_*p*_^*2*^ = .09; 38–19 p/c: *F* (3, 196) = 16.00, *p* < .001, *η*_*p*_^*2*^ = .20; 19–9 p/c: *F* (3, 196) = 19.41, *p* < .001, *η*_*p*_^*2*^ = .23; 9–5 p/c: *F*(3, 196) = 19.97, *p* < .001, *η*_*p*_^*2*^ = .23; 5–2 p/c: *F* (3, 196) = 39.27, *p* < .001, *η*_*p*_^*2*^ = .38; 2–1 p/c: *F* (3, 196) = 13.44, *p* < .001, *η*_*p*_^*2*^ = .17; residuals: *F* (3, 196) = 23.67, *p* < .001, *η*_*p*_^*2*^ = .27]. For the sake of clarity and to ease readability, mean SF values per image cluster as well as post hoc comparison between clusters are reported in Table 7.

**Table 7:** Means and standard deviations (in parenthesis) of spatial frequency bands (SF) per emotion-related group, and significant differences between clusters

SF bands	Disgust	Fear	Neither	Happiness
300–150 (p/c)	3.18 × 10^2^ (2.02 × 10^2^) ^a**^	4.85 × 10^2^ (2.97 × 10^2^) ^c*^	3.88 × 10^2^ (2.53 × 10^2^)	3.40 × 10^2^ (1.49 × 10^2^)
150–75 (p/c)	1.80 × 10^3^ (1.10 × 10^3^) ^a*^	2.56 × 10^3^ (1.32 × 10^3^)	2.34 × 10^3^ (1.51 × 10^3^)	2.33 × 10^3^ (1.07 × 10^3^)
75–38 (p/c)	8.85 × 10^3^ (5.54 × 10^3^) ^ab*c***^	1.29 × 10^4^ (6.95 × 10^3^)	1.23 × 10^4^ (6.41 × 10^3^)	1.46 × 10^4^ (7.25 × 10^3^)
38–19 (p/c)	4.62 × 10^4^ (2.99 × 10^4^) ^ab*c***^	6.77 × 10^4^ (3.86 × 10^4^) ^c***^	6.92 × 10^4^ (2.60 × 10^4^) ^c***^	9.98 × 10^4^ (5.57 × 10^4^)
19–9 (p/c)	2.32 × 10^5^ (1.87 × 10^5^) ^b**c***^	3.17 × 10^5^ (1.93 × 10^5^) ^c***^	3.76 × 10^5^ (1.70 × 10^5^) ^c***^	5.74 × 10^5^ (3.38 × 10^5^)
9–5 (p/c)	1.40 × 10^6^ (1.40 × 10^6^) ^bc***^	1.53 × 10^6^ (1.10 × 10^6^) ^bc***^	2.59 × 10^6^ (1.37 × 10^6^)	3.30 × 10^6^ (1.67 × 10^6^)
5–2 (p/c)	4.08 × 10^6^ (3.60 × 10^6^) ^bc***^	5.12 × 10^6^ (3.67 × 10^6^) ^bc***^	1.18 × 10^7^ (6.08 × 10^6^) ^c***^	1.66 × 10^7^ (1.04 × 10^7^)
2–1 (p/c)	4.98 × 10^6^ (5.30 × 10^6^) ^b**c***^	6.18 × 10^6^ (9.22 × 10^6^) ^c***^	1.12 × 10^7^ (1.04 × 10^7^)	1.58 × 10^7^ (1.16 × 10^7^)
Residuals (p/c)	1.36 × 10^7^ (1.79 × 10^7^) ^bc***^	2.07 × 10^7^ (2.15 × 10^7^) ^bc***^	5.37 × 10^7^ (4.97 × 10^7^) ^c*^	7.76 × 10^7^(6.22 × 10^7^)

SF- Spatial frequency; p/c- pixels per degree

^a^Significant difference with the fear-related group

^b^Significant difference with the neither-related group

^c^Significant difference with the happiness-related group

* *p* < .05; ** *p* < .01; *** *p* < .001

Table SM-3, available from the project web page on the Open Science Framework platform (https://osf.io/b5zs7), presents the values for luminosity, RMS and effective contrasts, chromatic complexity, and SF distribution per image.

##### Correlations between valence and arousal and the stimulus’ low-order visual properties

To examine possible associations between the valence and arousal and the computed low-order visual properties (i.e., luminosity, RMS and effective contrast, chromatic complexity, and SF), bivariate Pearson correlations were conducted.

Valence was positively correlated with luminosity, RMS contrast, and chromatic complexity. Specifically, the higher these low-order visual properties are, the more positive the stimuli were rated. In turn, arousal showed a significant positive correlation with effective contrast, meaning that the higher the stimulus’ effective contrast, the higher the arousal ratings. Lastly, spatial frequency distribution correlated positively with valence in several low and high-frequency bands (i.e., bands 75–38 p/c, 38–19 p/c, 19–9 p/c, 9–5 p/c, 5–2 p/c, 2–1 p/c, and residuals), while with arousal it was just found a positive correlation with a low-frequency band (i.e., 300–150 p/c) and two negative correlations with a high-frequency band (i.e., band 5–2 p/c) and with residuals. Table 8 displays Pearson’s *r* and *p* values for all the examined correlations.

**Table 8:** Results from the Pearson correlations between mean valence, arousal, luminosity, RMS and effective contrast, chromatic complexity, and the spectral density of the nine analyzed frequency bands

	Valence	Arousal
Luminosity		
*r*	.498	-.134
*p*	**<.001**	.058
RMS contrast		
*r*	.201	.103
*p*	**.004**	.145
Effective contrast		
*r*	.113	.177
*p*	.110	**.012**
Chromatic complexity		
*r*	.253	-.025
*p*	**<.001**	.728
SF 300–150 (p/c)		
*r*	-.090	.161
*p*	.205	**.023**
SF 150–75 (p/c)		
*r*	.060	.131
*p*	.396	.064
SF 75–38 (p/c)		
*r*	.223	.127
*p*	**.002**	.073
SF 38–19 (p/c)		
*r*	.398	.119
*p*	**<.001**	.094
SF 19–9 (p/c)		
*r*	.491	.061
*p*	**<.001**	.390
SF 9–5 (p/c)		
*r*	.534	-.112
*p*	**<.001**	.114
SF 5–2 (p/c)		
*r*	.627	-.152
*p*	**<.001**	**.031**
SF 2–1 (p/c)		
*r*	.404	-.084
*p*	**<.001**	.239
SF Residuals (p/c)		
*r*	.524	-.156
*p*	**<.001**	**.028**

*r* - the correlation coefficient; *p* - probability; *SF*- spatial frequency; p/c- pixels per cycle. Significant p values are in bold

## General discussion

The OBNIS image set, originally validated for a Japanese population (Shirai & Watanabe, 2022), consists of a selection of 200 images aiming to provide a comprehensive animal database for investigating negative emotions (i.e., disgust and fear) elicited visually. The goal of the present article was to validate the colored version of OBNIS’s images for a European Portuguese population, also examining possible cross-cultural differences between the Japanese and the Portuguese populations. Two studies were conducted: Study 1 replicated the methodology from the original article (see study 1 from Shirai & Watanabe, 2022) to allow a direct comparison between the two populations; Study 2 extended the exploration of the image set properties for the Portuguese population, examining whether the OBNIS’s images elicit emotions other than fear and disgust. In study 2, the low-order visual properties of stimuli critical for affective and cognitive research were also explored.

Identical to the Japanese population (Shirai & Watanabe, 2022), a 3 clusters solution was found in study 1, grouping images into disgust, fear, and neither-related groups. Notably, when just 3 emotional descriptors were considered, only 12% of the images were categorized in distinct clusters by the two populations. This indicates a high agreement between Portuguese and Japanese populations regarding the emotions elicited by OBNIS’s images.

However, despite the emotional descriptor associated with each image, remarkable differences between populations were observed when valence and arousal ratings were inspected. Japanese data (Shirai & Watanabe, 2022) evidence that, generally, participants rated OBNIS images as more negative than Portuguese participants and negatively valenced images as more arousing. In turn, Portuguese participants assessed positively valenced images as more arousing when compared to the Japanese population. These differences become even more apparent when the relation between valence and arousal ratings is examined. Arousal ratings obtained from Portuguese participants are an asymmetric V-shaped function of valence ratings (similar to the observed for other emotional image sets; e.g., Lang, 1994; Soares et al., 2015). In other words, although arousal is generally higher for negatively valenced stimuli, it increases with similar magnitude for both negatively and positively valenced stimuli. However, the data from the Japanese population only shows a similar trend for negatively valenced images. When positively valenced stimuli are considered, arousal was nearly independent (i.e., a flat slope) of valence. These cross-cultural differences support the notion that cultures have an important influence on the way individuals perceive, feel and express emotions (see Mesquita & Walker, 2003). Even more interesting is the fact that these differences are especially evident for positive valenced stimuli. From an evolutionary perspective, the evaluation of a given stimulus as negative or positive is thought to be related to the activation of a defensive or an appetitive motivational system (respectively), which, in turn, results in withdrawal or approach tendencies (Bradley et al., 2001; Bradley & Lang, 1994; Sakaki et al., 2012). The observed differences between populations indicate that, perhaps due to survival reasons, the activation of the defensive system and the arousal it elicits may be more automatic and therefore less prone to social and cultural influences than the appetitive system – where the differences between the two populations are more evident. However, which these cultural differences are and whether they are general or just related to the OBNIS stimuli remains a subject to be explored in future research. Notably, the observed differences stress also the need to assess tools like OBNIS in different cultures (Soares et al., 2015) and, ideally, with the participants of each study (e.g., participants assess the selected pictures at the end of the experiment) for better control (for a similar argument, see Carretié et al., 2019).

In study 2, we explored whether this increased arousal for positive valenced stimuli is related to Portuguese participants associating OBNIS stimuli with positive emotions such as happiness. Hence, instead of employing just three classification options as in study 1 and in the original validation (i.e., fear, disgust, and neither; Shirai & Watanabe, 2022), we used six basic emotions (Ekman, 1992) plus a “neither” option in study 2 to examine whether the stimuli could elicit other emotions besides fear and disgust. Remarkably, instead of the three image groups described in Shirai and Watanabe (2022) and in study 1, we found a four-group solution (disgust-related, fear-related, happiness-related, and neither-related groups). The “neither group” from study 1 was divided into a “neither-related group” (i.e., images that were not associated with either of the six basic emotions) and a “happiness-related group” including positive images. The images' valence and arousal ratings support this distinctiveness of a “happiness-related group”. Regarding valence, the images in the happiness-related group were rated in the second half of the scale (ranging from extremely negative to extremely positive) and significantly more positively than the images in the neither-related group. These, in turn, were rated around the scale's midpoint and significantly less negative than the disgust and fear-related groups (rated primarily on the first half of the scale). Regarding the arousal ratings, the images in the happiness-related group were rated as significantly more arousing than the images in the neither-related group, which, in turn, were rated as less arousing than the images in the disgust and fear-related groups. Hence, images in the happiness-related group seem to be distinct from those in the neither-related group, being associated with a positive and arousing emotional state. This point is especially relevant for future research. A substantial number of images initially classified as “neither” were associated with a positive mood, being as arousing as images in other emotion-related groups (i.e., disgust-related) in the Portuguese populations. Future research using the OBNIS’s images should consider this aspect to avoid possible confusion. Nevertheless, such a characteristic expands OBNIS applicability, making it a relevant tool for studies focused on fear and disgust and for research exploring positive emotional states (i.e., happiness).

An important aspect to consider is why only four emotional groups of images emerged when seven emotional descriptors were given to the participants, with more than 90% of the images (181 out of 200) being mainly associated with the emotion most represented in the group they were included. We speculate that this is related to how the image set was developed. Images in OBNIS were selected to induce disgust, fear, or neither (see Shirai & Watanabe, 2022). As expected, these three groups emerged in the cluster analysis. Nevertheless, a substantial number of images selected to belong to the neither-related group seem to have induced positive affect in the Portuguese participants. Importantly, only one of the basic emotions used as emotion descriptors (i.e., anger, fear, disgust, sadness, surprise, and happiness; Ekman, 1992) was positive, leading to the emergence of a fourth cluster, i.e., the happiness-related group.

Another important aspect of study 2 is the information provided regarding the low-order visual properties of the images constituting the OBNIS database. The several low-order visual properties computed here (i.e., luminosity, RMS and effective contrasts, chromatic complexity, and SF distribution) present significant differences between distinct image groups and, in some cases, directly correlate with valence and arousal ratings. These visual properties have also been shown to interact with the images’ affective value in other databases (Carretié et al., 2019; Delplanque et al., 2007). This is congruent with several studies showing, for instance, that brighter images are rated as more positive (Lakens et al., 2013), that SF content modulates the processing of fear-related stimuli (Gomes et al., 2018), images with higher chromatic complexity elicit greater occipital positive event-related potentials (Bradley et al., 2007), and emotional scenes tend to present greater spectral densities on low SF bands when compared to neither ones (Delplanque et al., 2007). Hence, controlling such low-order visual properties is crucial for researchers who want to isolate the effects of the affective content of images independently of their physical characteristics. The values computed in this paper may be used to select a set of pictures controlled for a given set of visual properties. Another possible approach is to use specific software (Willenbockel et al., 2010) to equalize the stimuli's visual properties. However, another assessment of the images’ affective value after the equalization should be conducted because how these controlling processes affect their emotional content is still open.

An important limitation of the present research is that our sample, in study 2, is not gender-balanced. Research using other databases (Bradley, Codispoti, Sabatinelli, et al., 2001) reported that gender differences regarding arousal and valence ratings of emotional pictures are common. In study 1, females rated negatively valenced images as more negative and positively valenced images as more arousing than males. Whether these differences in arousal and valence ratings would be also reflected in gender differences in the discrimination between distinct emotions (with special emphasis on the ‘happiness’ image group) remains an open question to be addressed in future research. Moreover, future studies aiming to increase OBNIS applicability should consider other cultures (as previously mentioned) and other age groups since there is research evidencing that emotional processing may change with age (Urry & Gross, 2010). Hence, ratings obtained with a university population may not apply, for instance, to older adults.

In conclusion, this OBNIS validation for a Portuguese population provides the emotional classification and valence and arousal ratings per image for a different population than the one used in the original study, highlighting potential cross-cultural differences that should be taken into account when one aims to use this, and other, image sets. It also includes an exploration of the visual low-order properties that may affect the emotional processing of the images. From our perspective, this image set constitutes a relevant tool for, among others, studies exploring the role of fear and disgust in several anxiety-related pathologies (e.g., Cisler et al., 2009), or attentional studies comparing affective animal and non-animal stimuli (see Cosmides & Tooby, 2007). This article is designed to increase the utility of this relevant image set for studies aiming to induce disgust, fear, or (at least in Portuguese participants) happiness using biological stimuli not comprising visually complex scenes that are harder to control for their content and physical properties. From our results, it is possible to argue that for some populations, the OBNIS (Open Biological Negative Image Set) is instead the Open Biological Negative and Positive Image Set.

## Data Availability

The image set originally created by Shirai and Watanabe (2022), i.e., the 500 × 500 px version of the images, as well as the images' grayscale version, can be found and freely downloaded on the authors’ OSF website: https://osf.io/pfrx4/?view_only=911b1be722074ad4aab87791cb8a72f5. The colored images resized to an area of 300 by 300 pixels used in the present validation study can be found at: https://osf.io/b5zs7. The data from both studies are available at https://osf.io/b5zs7. Study 2 was preregistered at https://osf.io/75evj.

## Footnotes

1 As revealed by a Kruskal–Wallis non-parametric test [*χ*^*2*^_*KW*_(3) = .95, *p* = .814], no significant statistical differences were observed regarding the number of assessments between the images constituting the distinct emotion-related group (see the section Emotion classification below).

## Code availability

The analysis code is available at https://osf.io/b5zs7.

## References

[CR1] Adobe Inc. (2012). Adobe Photoshop (CS6). Retrieved from https://www.adobe.com/products/photoshop.html

[CR2] Alarcão SM, Fonseca MJ (2018). Identifying emotions in images from valence and arousal ratings. Multimedia Tools and Applications.

[CR3] Barrett LF (2012). Emotions are real. Emotion.

[CR4] Barrett LF, Russell JA (1999). The structure of current affect. Current Directions in Psychological Science.

[CR5] Becker ES, Rinck M, Türke V, Kause P, Goodwin R, Neumer S, Margraf J (2007). Epidemiology of specific phobia subtypes: Findings from the dresden mental health study. European Psychiatry.

[CR6] Bradley MM, Lang PJ (1994). Measuring emotion: The self-assessment manikin and the semantic differential. Journal of Behavior Therapy and Experimental Psychiatry.

[CR7] Bradley MM, Codispoti M, Cuthbert BN, Lang PJ (2001). Emotion and motivation I: Defensive and appetitive reactions in picture processing. Emotion.

[CR8] Bradley MM, Hamby S, Löw A, Lang PJ (2007). Brain potentials in perception: picture complexity and emotional arousal. Psychophysiology.

[CR9] Cacioppo JT, Berntson GG (1994). Relationship between attitudes and evaluative space: A critical review, with emphasis on the separability of positive and negative substrates. Psychological Bulletin.

[CR10] Carretié L, Tapia M, López-Martín S, Albert J (2019). EmoMadrid: An emotional pictures database for affect research. Motivation and Emotion.

[CR11] Charrad M, Ghazzali N, Boiteau V, Niknafs A (2014). NbClust: An R Package for Determining the Relevant Number of Clusters in a Data Set. Journal of Statistical Software.

[CR12] Cisler JM, Olatunji BO, Lohr JM (2009). Disgust, fear, and the anxiety disorders: a critical review. Clinical Psychology Review.

[CR13] Ćoso B, Guasch M, Ferré P, Hinojosa JA (2019). Affective and concreteness norms for 3,022 Croatian words. Quarterly Journal of Experimental Psychology.

[CR14] Dan-Glauser ES, Scherer KR (2011). The Geneva affective picture database (GAPED): A new 730-picture database focusing on valence and normative significance. Behavior Research Methods.

[CR15] Davidson RJ, Cacioppo JT (1992). New developments in the scientific study of emotion: An introduction to the special section. Psychological Science.

[CR16] Delplanque S, N’diaye K, Scherer K, Grandjean D (2007). Spatial frequencies or emotional effects? A systematic measure of spatial frequencies for IAPS pictures by a discrete wavelet analysis. Journal of Neuroscience Methods.

[CR17] Ekman P (1992). Are there basic emotions?. Psychological Review.

[CR18] Field A (2014). Discovering Statistics Using SPSS.

[CR19] Funke F, Reips U-D (2012). Why semantic differentials in web-based research should be made from visual analogue scales and not from 5-point scales. Field Methods.

[CR20] Gayet, S., Stein, T., & Peelen, M. V. (2019). The danger of interpreting detection differences between image categories: A brief comment on “mind the snake: fear detection relies on low spatial frequencies” (Gomes, Soares, Silva, & Silva, 2018). In *Emotion*. 10.1037/emo0000550 10.1037/emo0000550 30762381

[CR21] Gomes N, Soares SC, Silva S, Silva CF (2018). Mind the snake: Fear detection relies on low spatial frequencies. Emotion.

[CR22] Gomes N, Silva S, Soares SC (2019). “Threat-unrelated” properties: An ill-defined concept. A reply to “The danger of interpreting detection differences between image categories” (Gayet, Stein, & Peelen, 2019). Emotion.

[CR23] Gray JA (1987). *The psychology of fear and stress*.

[CR24] Grimaldos, J., Duque, A., Palau-Batet, M., Pastor, M. C., Bretón-López, J., & Quero, S. (2021). Cockroaches are scarier than snakes and spiders: Validation of an affective standardized set of animal images (ASSAI). *Behavior Research Methods*. 10.3758/s13428-021-01577-7 10.3758/s13428-021-01577-7 PMC8025455 33826093

[CR25] Holmes KJ, Lourenco SF (2011). Common spatial organization of number and emotional expression: A mental magnitude line. Brain and Cognition.

[CR26] JASP Team. (2022). *JASP* (0.16.2). https://jasp-stats.org/

[CR27] Jiang Y, Costello P, He S (2007). Processing of invisible stimuli: Advantage of upright faces and recognizable words in overcoming interocular suppression. Psychological Science.

[CR28] John CH (1988). Emotionality ratings and free-association norms of 240 emotional and non-emotional words. Cognition & Emotion.

[CR29] Johnson DE (1998). *Applied Multivariate Methods for Data Analysts*.

[CR30] Kuppens P, Tuerlinckx F, Russell JA, Barrett LF (2013). The relation between valence and arousal in subjective experience. Psychological Bulletin.

[CR31] Kurdi B, Lozano S, Banaji MR (2017). Introducing the open affective standardized image set (OASIS). Behavior Research Methods.

[CR32] Kurt P, Eroğlu K, Bayram Kuzgun T, Güntekin B (2017). The modulation of delta responses in the interaction of brightness and emotion. International Journal of Psychophysiology.

[CR33] Lakens D, Fockenberg DA, Lemmens KPH, Ham J, Midden CJH (2013). Brightness differences influence the evaluation of affective pictures. Cognition & Emotion.

[CR34] Lang PJ, van Goozen SHM, Van de Poll NE, Sergeant JA (1994). The motivational organization of emotion: Affect-reflex connections. *Emotions: Essays on emotion theory*.

[CR35] Lang PJ, Bradley MM, Cuthbert BN (2005). *International affective picture system (IAPS): Technical manual and affective ratings*.

[CR36] Lohani M, Gupta R, Srinivasan N (2013). Cross-cultural evaluation of the international affective picture system on an Indian sample. Psychological Studies.

[CR37] Marchewka A, Żurawski Ł, Jednoróg K, Grabowska A (2014). The Nencki Affective Picture System (NAPS): Introduction to a novel, standardized, wide-range, high-quality, realistic picture database. Behavior Research Methods.

[CR38] Matchett G, Davey GCL (1991). A test of a disease-avoidance model of animal phobias. Behaviour Research and Therapy.

[CR39] Matejka, J., Glueck, M., Grossman, T., & Fitzmaurice, G. (2016). The Effect of Visual Appearance on the Performance of Continuous Sliders and Visual Analogue Scales. *Proceedings of the 2016 CHI Conference on Human Factors in Computing Systems*, 5421–5432. 10.1145/2858036.2858063

[CR40] Mattek AM, Wolford GL, Whalen PJ (2017). A Mathematical Model Captures the Structure of Subjective Affect. Perspectives on Psychological Science.

[CR41] Mesquita B, Walker R (2003). Cultural differences in emotions: A context for interpreting emotional experiences. Behaviour Research and Therapy.

[CR42] Müller MM, Andersen SK, Keil A (2008). Time course of competition for visual processing resources between emotional pictures and foreground task. Cerebral Cortex (New York, N.Y. : 1991).

[CR43] Murtagh F, Legendre P (2014). Ward’s hierarchical agglomerative clustering method: Which algorithms implement ward’s criterion?. Journal of Classification.

[CR44] Öhman A, Mineka S (2001). Fears, phobias, and preparedness: Toward an evolved module of fear and fear learning. Psychological Review.

[CR45] Öhman A, Mineka S (2003). The malicious serpent: Snakes as a prototypical stimulus for an evolved module of fear. Current Directions in Psychological Science.

[CR46] Olatunji BO, Sawchuk CN (2005). Disgust: Characteristic features, social manifestations, and clinical implications. Journal of Social and Clinical Psychology.

[CR47] Peirce J, Gray JR, Simpson S, MacAskill M, Höchenberger R, Sogo H, Kastman E, Lindeløv JK (2019). PsychoPy2: Experiments in behavior made easy. Behavior Research Methods.

[CR48] Pitt B, Casasanto D, Grodnerd D, Mirman D, Papafragou A, Trueswell J (2016). Spatializing Emotion: A Mapping of Valence or Magnitude?. *Proceedings of the 38th Annual Conference of the Cognitive Science Society*.

[CR49] Polák J, Rádlová S, Janovcová M, Flegr J, Landová E, Frynta D (2020). Scary and nasty beasts: Self-reported fear and disgust of common phobic animals. British Journal of Psychology.

[CR50] R Core Team. (2022). *R: A language and environment for statistical computing *(4.2.2). R Foundation for Statistical Computing. https://www.r-project.org/

[CR51] Raftery AE (1995). Bayesian Model Selection in Social Research. Sociological Methodology.

[CR52] Redies C, Grebenkina M, Mohseni M, Kaduhm A, Dobel C (2020). Global Image Properties Predict Ratings of Affective Pictures. Frontiers in Psychology.

[CR53] Rozin P, Fallon AE (1987). A perspective on disgust. Psychological Review.

[CR54] Sakaki M, Niki K, Mather M (2012). Beyond arousal and valence: The importance of the biological versus social relevance of emotional stimuli. Cognitive, Affective, & Behavioral Neuroscience.

[CR55] Sharma, S. (1996). *Applied Multivariate Techniques*. Wiley.

[CR56] Shirai R, Watanabe K (2022). Open biological negative image set. Royal Society Open Science.

[CR57] Soares AP, Pinheiro AP, Costa A, Frade CS, Comesaña M, Pureza R (2015). Adaptation of the International Affective Picture System (IAPS) for European Portuguese. Behavior Research Methods.

[CR58] Sowden PT, Schyns PG (2006). Channel surfing in the visual brain. Trends in Cognitive Sciences.

[CR59] Staňková H, Janovcová M, Peléšková Š, Sedláčková K, Landová E, Frynta D (2021). The ultimate list of the most frightening and disgusting animals: Negative emotions elicited by animals in Central European respondents. Animals.

[CR60] Strauss GP, Allen DN (2008). Emotional intensity and categorisation ratings for emotional and nonemotional words. Cognition and Emotion.

[CR61] Susskind JM, Lee DH, Cusi A, Feiman R, Grabski W, Anderson AK (2008). Expressing fear enhances sensory acquisition. Nature Neuroscience.

[CR62] t Hart BM, Schmidt HCEF, Klein-Harmeyer I, Einhäuser W (2013). Attention in natural scenes: contrast affects rapid visual processing and fixations alike. Philosophical Transactions of the Royal Society B: Biological Sciences.

[CR63] Tabachnick BG, Fidell LS (1996). *Using multivariate statistics*.

[CR64] Tsai JL, Knutson B, Fung HH (2006). Cultural variation in affect valuation. Journal of Personality and Social Psychology.

[CR65] Urry HL, Gross JJ (2010). Emotion Regulation in Older Age. Current Directions in Psychological Science.

[CR66] Vimal RLP (2002). Spatial frequency discrimination: a comparison of achromatic and chromatic conditions. Vision Research.

[CR67] Webb K, Davey GCL (1992). Disgust sensitivity and fear of animals: Effect of exposure to violent or revulsive material. Anxiety, Stress & Coping.

[CR68] Willenbockel V, Sadr J, Fiset D, Horne GO, Gosselin F, Tanaka JW (2010). Controlling low-level image properties: the SHINE toolbox. Behavior Research Methods.

[CR69] Yao Z, Wu J, Zhang Y, Wang Z (2017). Norms of valence, arousal, concreteness, familiarity, imageability, and context availability for 1,100 Chinese words. Behavior Research Methods.

[CR70] Yik M, Russell JA, Steiger JH (2011). A 12-point circumplex structure of core affect. Emotion.

[CR71] Yik, M., Mues, C., Sze, I. N. L., Kuppens, P., Tuerlinckx, F., De Roover, K., … Russell, J. A. (2022). On the relationship between valence and arousal in samples across the globe. *Emotion*. 10.1037/emo0001095 10.1037/emo0001095 35446055

